# Linking spatial drug heterogeneity to microbial growth dynamics in theory and experiment

**DOI:** 10.1371/journal.pcbi.1013896

**Published:** 2026-01-20

**Authors:** Zhijian Hu, Yuzhen Wu, Tomas Freire, Erida Gjini, Kevin Wood

**Affiliations:** 1 Department of Biophysics, University of Michigan, Ann Arbor, Michigan, United States of America; 2 Department of Mathematics, University of Michigan, Ann Arbor, Michigan, United States of America; 3 Center for the Study of Complex Systems, University of Michigan, Ann Arbor, Michigan, United States of America; 4 Department of Molecular, Cellular, and Developmental Biology, University of Michigan, Ann Arbor, Michigan, United States of America; 5 Center for Computational and Stochastic Mathematics, Instituto Superior Técnico, University of Lisbon, Lisbon, Portugal; 6 Department of Physics, University of Michigan, Ann Arbor, Michigan, United States of America; University of Connecticut School of Medicine, UNITED STATES OF AMERICA

## Abstract

Drugs play a central role in limiting bacterial population spread, yet laboratory studies typically assume well-mixed environments when assessing microbial drug responses. In contrast, bacteria in the human body often occupy spatially structured habitats where drug concentrations vary. Understanding how this heterogeneity shapes growth and decline is therefore essential for controlling infections and mitigating resistance evolution. Here, we developed a minimal robot-automated system to study how spatial drug heterogeneity affects short-term population dynamics in *E. faecalis*, a Gram-positive opportunistic pathogen. This system was combined with a theoretical framework to interpret and explain the observed outcomes. We first recapitulated the classic critical-patch-size model result: in a spatially homogeneous environment, a population persists in a finite domain only when growth outpaces diffusive losses at the boundaries. In heterogeneous environments, we found certain conditions that population persistence can depend critically on the spatial arrangement of the drug, even when its total amount is fixed. Using theoretical and experimental approaches, we identified the arrangements that produce the strongest growth and the fastest decline, revealing the range of possible outcomes under drug heterogeneity. We further tested this framework in more complex environments, including ring-shaped communities, and observed consistent arrangement-dependent behavior. Overall, our results extend the classical growth-condition framework to general heterogeneous environments and demonstrate that spatial drug arrangement - not only total dose - can strongly influence bacterial population dynamics. These findings highlight the importance of spatially structured dosing strategies and motivate further theoretical and experimental investigation.

## Author summary

Understanding how bacteria respond, grow and spread in the presence of antibiotics is crucial for fighting infections and preventing drug resistance. In many real-world environments, like the human body, antibiotics are not spread evenly. Instead, they often create a patchwork of high- and low-drug regions. In this study, we built a simple lab system that mimics this kind of uneven landscape, using a common bacterium existing in human’s gut and a robotic platform. We found that the overall outcome, whether the bacterial population grows or dies out, depends not just on how much drug is present, but on how and where exactly the drug is placed. Even with the same total drug amount, different spatial layouts led to very different results. Using computer simulations and mathematical tools, we showed that this effect could be predicted and explained. Our findings highlight the importance of drug distribution in treatment strategies and suggest that how antibiotics are arranged in space may be just as important as how much is used.

## Introduction

Antibiotics are widely used to control bacterial infections [1,2]. However, the environments in which bacterial populations live, especially within the human body, are often spatially structured and heterogeneous [3–6]. Previous studies have primarily examined how spatial drug heterogeneity shapes long-timescale resistance evolution [6–23]. Under most conditions, monotonic spatial gradients in drug concentration dramatically accelerate resistance evolution [7–17]. More recently, theoretical work has highlighted that non-monotonic spatial profiles can generate qualitatively different evolutionary outcomes [20,21], showing that depending on the interplay between growth, migration, and mutation, spatial drug heterogeneity can either accelerate or decelerate evolution. Together, these findings underscore the complexity of how bacterial populations respond to spatially heterogeneous drugs over evolutionary timescales.

Despite this progress, our understanding of ecological-timescale dynamics under spatial drug heterogeneity remains limited, largely due to the lack of appropriate data. Laboratory studies typically measure dose–response curves in well-mixed conditions [24–32], and extensions to controlled spatially heterogeneous environments are rare. Clinically, treatment-induced resistance [33] often arises from ecological-scale selective pressures and incomplete bacterial clearance, well before resistance mutations emerge [34]. Thus, understanding short-term population responses, such as population increase, decline, or clearance, in spatially heterogeneous drug landscapes is a critical first step toward preventing resistance emergence. As in evolutionary contexts, these ecological responses may also diverge depending on spatial drug arrangements, though this has not been systematically explored.

Although not originally motivated by drug heterogeneity, spatial ecology offers a classical framework for understanding growth in structured environments. The “critical patch size” condition, derived from the reaction–diffusion KiSS (Kierstead–Slobodkin–Skellam) model [35,36], states that a population in a confined habitat can persist only if the domain is sufficiently large for intrinsic growth to outweigh diffusive losses to absorbing boundaries. Several theoretical extensions have examined heterogeneous growth rates or diffusion coefficients, showing that heterogeneity shifts this threshold by modifying the principal eigenvalue of the growth–diffusion operator [37–39]. For heterogeneous growth landscapes, specific examples demonstrate that different arrangements of high- and low-growth regions can either strengthen or weaken persistence, effectively altering the patch-size condition [37]. Heterogeneous diffusion has been shown to generally reduce the critical patch size [40]. Experimentally, *E. coli* growth under uniform UV-light habitats provides evidence of the critical patch-size effect [41], and a related “oasis–desert” landscape experiment has demonstrated that spatial structure influences population growth and collapse [42]. However, these results are not drug-related, and they represent isolated examples rather than systematic investigations across different spatial patterns or parameters such as diffusion, in part due to the labor-intensive nature of such experiments. Theoretically, prior work provides specific case studies but lacks a general discussion of when arrangement dependence emerges and what spatial arrangements most strongly promote growth or decline.

Building on the KiSS framework, we developed a minimal experimental system automated by a pipetting robot, to study how spatial distributions of drug concentration in a one-dimensional confined environment influence short-term bacterial responses. Using *Enterococcus faecalis*, a Gram-positive opportunistic pathogen common in the human gastrointestinal tract and responsible for infections such as infective endocarditis and urinary tract infections [43–47], we first recapitulated the classical critical patch-size condition by performing parameter sweeps over patch sizes and drug-induced growth rates in spatially homogeneous environments. We then investigated heterogeneous environments by fixing the total drug amount and designing diverse spatial arrangements. We found that whether the population persists or declines depends sensitively on the specific spatial arrangement of drug. Extending the classical framework, we identified the general condition under which such “arrangement dependence” arises, and the conditions under which population responses remain arrangement-independent, using theoretical optimization and experimental parameter sweeps. Our results further reveal two optimal spatial arrangements that generate the most extreme outcomes: a central high-growth (drug-free) region maximizes population growth, whereas a central low-growth (saturated drug concentration) region maximizes population decline. All other arrangements fall between these two extremes. This pattern can be explained analytically using perturbation approximations of the growth condition. Extending the approach to more complex spatial structures beyond the confined framework, such as ring-shaped communities, further confirms the importance of spatial arrangement.

Overall, our study establishes a direct link between theoretical predictions and experimental measurements of bacterial population dynamics in spatially heterogeneous drug environments. It extends the classical homogeneous growth-condition framework to general heterogeneous settings and demonstrates that spatial arrangement - not just the total amount of drug - can profoundly influence bacterial growth outcomes.

## Methods

### Theoretical framework

Here we use the classic one-dimensional Fisher–KPP model [48,49], linearized at low population densities with two Dirichlet (absorbing) boundaries, as our theoretical framework. In spatial ecology this is also known as the KiSS (Kierstead–Slobodkin–Skellam) model [35,36]:

$$\frac{\partial u}{\partial t}=\beta \frac{\partial^{2}u}{\partial x^{2}}+g(D(x))u,$$ (1)

**Fig 1:**
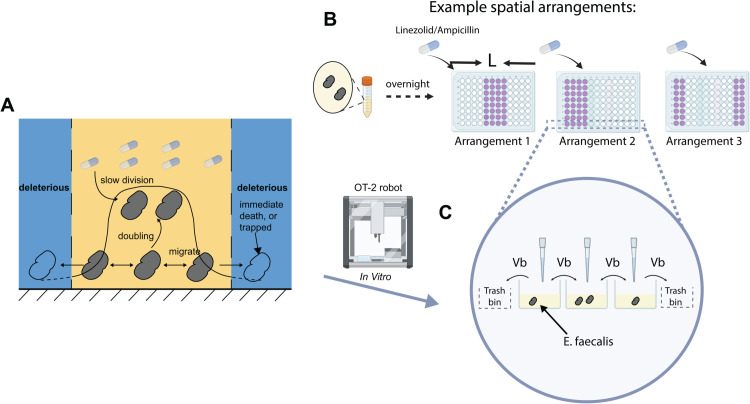
Schematic of growth-migration dynamics in a deleteriously confined environment under spatial drug heterogeneity, and corresponding experimental design. **A.** The illustration represents the bacteria population (the grey icon) proliferating and migrating in a deleteriously confined environment under spatial drug heterogeneity (the blue-white drug capsules). **B, C** The *E. faecalis* was grown overnight and then diluted 1:1 into different 96-well plates with a spatially uniform population density profile, but different drug concentrations *D* and spatial arrangements, at initial time *T* = 0. In the illustration, purple wells represent wells with the saturated drug concentration, while white wells represent drug-free wells. After every growth cycle of Δt h, bacteria migration was performed by transferring the same amounts *Vb* of bacteria liquid to both neighboring wells along the columns by the OT-2 pipetting robot; *V* is the total volume per well and usually is 200μL; b is the transferred fraction. Bacteria at 2 boundary wells were taken out at the same volume *Vb*. Cycles would be repeated. Usually it’s ∼8 times. Cell density profiles were measured by plate reader exactly before the next migration/volume transfer. The clip-art icons of the pipette tip, drug capsule, tube, 96-well plate and OT-2 pipetting robot were created in BioRender. Hu, Z. (2025) https://BioRender.com/zq812ma.

Here, *u* is the cell density at position *x* and time *t*; *g*(*D*(*x*)) is the spatially varying growth rate determined by the local drug concentration *D*(*x*); *β* is the diffusion rate (although diffusion and migration differ in units, we use the two terms interchangeably in this paper for the benefit of a broader audience); and *L* is the size of the one-dimensional confined region (see S1 Text, Sect 1 for more details). The boundary conditions represent the edges of the confined habitat, outside of which are deleterious regions - bacteria die or are trapped immediately causing population loss (Fig 1A).

For short-term bacterial dynamics on ecological timescales, we focus on whether populations ultimately survive or go extinct. If we denote the growth–migration operator $g(D(x))\;+\;\beta \frac{\partial^{2}}{\partial x^{2}}$ as Ω, then the survival criterion is determined by the largest eigenvalue $\lambda_{0}$ of Ω, which represents the long-term growth rate after sufficient time (S1 Text, Sect 4 and Fig A):

$$\lambda_{0}=\|\Omega \|=\left\|g(D(x))+\beta \frac{\partial^{2}}{\partial x^{2}}\right\|.$$ (2)

Here ‖·‖ is won the sign of $\lambda_{0}$, which is determined by the interplay between the spatial growth term *g*(*D*(*x*)) and the migration term $\beta \frac{\partial^{2}}{\partial x^{2}}$.

**Fig 2:**
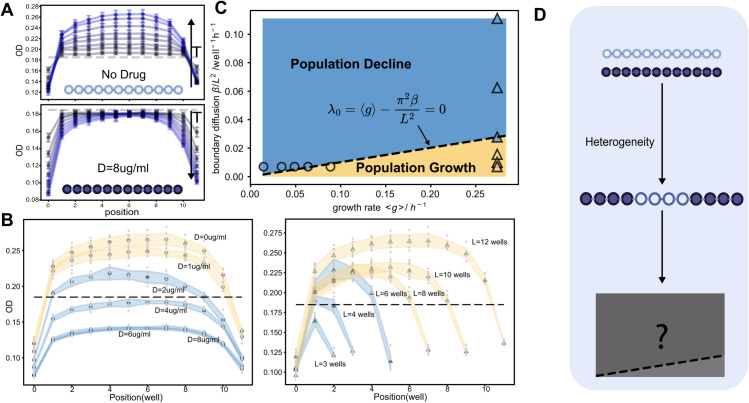
Bacteria population response (growth/decline) in different drug concentrations and migration regimes of a bacteriostatic antibiotic, Linezolid, for 8 cycles. **A.** Position-specific bacteria growing process under spatial drug homogeneity in drug-free(D=0 μg/mL) regimes and high-drug(D=8 μg/mL) regimes. At the bottom of each figure, a 12-well illustration with light or dark colors represents drug-free wells or drug wells. The dashed line in each figure is the initial spatial cell density at *T* = 0. Grey blue dots and curves are early cycles while light blues are late cycles. For drug-free regimes, as time increases, the curve is gradually shifting up while the spatial density curve is decreasing down for high-drug regimes. Each single curve with error bars (standard deviation) including initial cell densities are averaged over 8 technical replicates. **B.** Endpoint OD curves, for 6 different drug concentrations *D* (left panel, circles) and 6 system sizes *L* (right panel, triangles). Orange represents conditions where the final bacterial population increases, while blue indicates where the population decreases. The shaded regions denote error margins (standard deviation) calculated with 8 technical replicates shown as small-size grey dots. **C.** A phase diagram showing the relationship between the boundary diffusion effect, $\frac{\beta }{L^{2}}$, and the spatially averaged growth rate, ⟨g⟩. Circles and triangles are endpoint data points from Panel B. Blue or orange colors represent observed population decline or growth in experiments. **D.** An illustration shows that switching from spatial drug homogeneity to heterogeneity may change the growth–migration phase diagram shown in Panel C.

For homogeneous environments, $\lambda_{0}$ can be obtained analytically. The population goes extinct or shows a declining response when

$$\lambda_{0}=\langle g\rangle -\frac{\pi^{2}\beta }{L^{2}}<0,$$ (3)

where ⟨g⟩=g(D) is the uniform growth rate. Setting $\lambda_{0}=0$ yields

$$L_{c}=\pi \sqrt{\frac{\beta }{\left\langle g\right\rangle }},$$ (4)

which corresponds to the classical “critical patch size” in the KiSS model [35,36]. We will later show that we can experimentally recapitulate this result using our robot-automated system (Results subsection “Recapitulation of the classic growth condition of the critical-patch-size model under homogeneous environments”; and Fig 2).

For heterogeneous drug environments, there is no closed-form analytical expression for $\lambda_{0}$. Instead, we introduce i. a constrained optimization method to identify the “arrangement-dependent” condition under which survival depends on the specific spatial drug arrangement, and ii. a first-order perturbation approximation to describe how spatial heterogeneity contributes to $\lambda_{0}$ and why two extreme spatial arrangements exist (For more derivation details, see S1 Text, Sect 1.3 and 1.4). These results are discussed in the following Results subsection “Theory validates the indicated “arrangement-dependent” phase of mixed responses, and explains optimal spatial arrangements” and Fig 4.

### Experimental set-up for 1D minimal system automated by a pipetting robot

To study the spatial effects described in the theoretical framework, we used an OT-2 pipetting robot (Opentrons) to automate otherwise labor-intensive experimental procedures and improve controllability. *E. faecalis* populations were transferred along selected wells in a row of a 96-well plate to mimic a one-dimensional space (Fig 1B and 1C). The spatial-varying growth rate *g* is modulated by different drug concentrations in different wells. The system size *L* is set by the number of wells, and migration is implemented by exchanging small volumes *Vb* of media between adjacent wells, where *V* is the total well volume and *b* is the transferred fraction.

For boundary conditions, the same volume *Vb* was removed from the two edge wells to mimic diffusive loss into a deleterious environment. These wells were then replenished with either drug-free or high-drug media, depending on the spatial drug arrangement. Spatial growth rates were controlled using the bacteriostatic drug Linezolid. For experiments involving a ring-shaped (periodic) community, we used the bactericidal drug Ampicillin to induce population loss. Each experiment followed a growth–migration cycle: bacteria first grew for Δt hours, then migration was performed by pipetting, and the cycle was repeated. Additional experimental conditions and protocols are described in the S1 Text, Sect 2.1.

Importantly, the full experiment was restricted to a short duration (*T* = 2–4 hours). Thus, we observed population density increase or decline rather than true long-term survival or extinction. This short duration offers several advantages: i. de novo mutations and resistance evolution are negligible; ii. growth dynamics can be approximated by exponential models for analytical tractability; and iii. drug diffusion between wells is minimal (See S1 Text, Sect 5 for more discussions). Although short-term growth or decline is not strictly equivalent to long-term persistence or extinction, the two outcomes are effectively aligned. Therefore, we use these terms interchangeably in this paper (S1 Text, Sect 4). Growth or decline was determined by comparing final and initial optical density (OD) values at 600nm measured by an Enspire Multimodal Plate Reader (Perkin Elmer).

To relate experimental parameters to the model parameters *g* and *β* in Eq 1, we expressed the repeated growth–migration cycle as a discrete dynamical equation and discretized the model for comparison. This yields g=⟨g⟩, $\beta =b\frac{\Delta x^{2}}{\Delta t}\left(1+\langle g\rangle \Delta t\right),$ where Δx=1 well and Δt=0.25/0.5 h (S1 Text, Sect 2.2). A summary table of parameters used in both the model and experiments is provided in Table 1.

**Table 1:** Summary of model and experimental parameters used in this study.

Symbol	Name/Meaning	Value/Unit
*D*(*x*)	Drug concentration at position *x*	0 or 8 *μ*g/mL (Linezolid, Heterogeneous condition) 0 or 100 *μ*g/mL (Ampicillin, Periodic condition) For drug concentrations of Linezolid under homogeneous condition, see Fig 2
*g*(*D*)	Growth rate under drug concentration *D*	$g(0)=0.1645\;{\text{h}}^{-1}$; $g(8)=0.0724\;{\text{h}}^{-1}$ (Linezolid, Heterogeneous condition) $g(0)=0.1930\;{\text{h}}^{-1}$; $g(100)=-0.3827\;{\text{h}}^{-1}$ (Ampicillin, Periodic condition) For growth rates induced by Linezolid under homogeneous condition, see Fig 2
*b*	Volume transfer fraction per migration step	0.025–0.5 (dimensionless); see caption of Fig 5
*β*	Diffusion coefficient (effective migration)	$\beta \approx b\;\Delta x^{2}/\Delta t$; well^2^/hour
*L*	System size (total number of wells)	3–12 wells
$\lambda_{0}$	Largest eigenvalue of growth-migration operator	1/hour (sign determines growth or extinction)
δg	Spatial deviation of growth rate from mean	1/hour
⟨g⟩	Spatially averaged growth rate	1/hour
*V*	Total liquid volume per well	200 μL
$V_{b}$	Volume transferred per migration step	$V_{b}=bV$; *μ*L
Δt	Duration of one growth cycle	0.25 hour (Homogeneous condition) or 0.5 hour (Heterogeneous/Periodic condition)
*T*	Duration of experiment	2–4 hours (8 cycles per experiment; depends on Δt)

## Results

### Recapitulation of the classic growth condition of “critical-patch-size” model under homogeneous environments

For the homogeneous environment, we focus on the largest eigenvalue $\lambda_{0}=\langle g\rangle \;-\;\frac{\pi^{2}\beta }{L^{2}}$ from the KiSS model, which can be separated into two terms describing the uniform growth (⟨g⟩) and the boundary diffusion effect ($\frac{\beta }{L^{2}}$). The uniform growth rate drives the increase in population density, while the boundary diffusion term contributes to population loss. A natural question is whether the final responses observed in our experimental system match the model prediction based on the competition between uniform growth (⟨g⟩) and boundary diffusion ($\frac{\beta }{L^{2}}$).

By varying the uniform drug concentration *D*, we find that under low drug concentrations bacteria can grow despite the deleterious environment. As *D* increases, bacterial growth rate diminishes, weakening the population’s ability to counteract cell loss through boundary diffusion. Once reproduction can no longer balance this loss, the population shifts into decline, ultimately leading to extinction as density approaches zero in the long-time limit. Fig 2A shows bacterial growth in drug-free conditions (D=0 μg/ml) and at high drug concentrations (D=8 μg/ml). As drug concentration increases (Fig 2B, left panel), the collective spatial response shifts from growth to decline.

Next, to investigate the boundary diffusion effect $\frac{\beta }{L^{2}}$ directly, we fix the homogeneous growth rate (no drug) and vary the system size *L*. Consistent with model predictions, the population shifts from growth to decline as *L* decreases from 12 wells to 3 wells (Fig 2B, right panel). Our experimental data agrees with the phase diagram predicted by the KiSS model (Fig 2C), where the transition boundary is set by $\lambda_{0}=\langle g\rangle -\frac{\pi^{2}\beta }{L^{2}}=0$. Thus, our experimental system successfully recapitulates the classic “critical-patch-size” result under homogeneous environments.

### Different spatial drug arrangements modulate growth dynamics

In homogeneous drug environments, bacterial communities either grow or decline, determined solely by boundary diffusion and a fixed growth rate. However, in a spatially heterogeneous drug environment, different spatial arrangements may result in distinct growth dynamics and different population outcomes, even when the spatially averaged growth rate ⟨g⟩ is the same. Our next goal is to understand how spatial drug heterogeneity alters growth dynamics experimentally, and whether any emerging patterns can be predicted using our model framework (Fig 2D).

For a specific total drug amount, or equivalently a fixed spatially averaged drug concentration, many possible spatial drug arrangements can be constructed. For simplicity, we use two types of wells containing D=0 μg/mL and D=8 μg/mL to create heterogeneous “step-like” environments consisting of drug and drug-free wells. An advantage of this design is that a given total drug amount corresponds to a unique spatially averaged growth rate ⟨g⟩. The spatially averaged growth rate can therefore be represented by the number of drug wells *n*_*D*_, while keeping *n*_*D*_ fixed and permuting their order for comparison. Throughout this paper, we use total drug amount and spatially averaged growth rate interchangeably.

**Fig 3:**
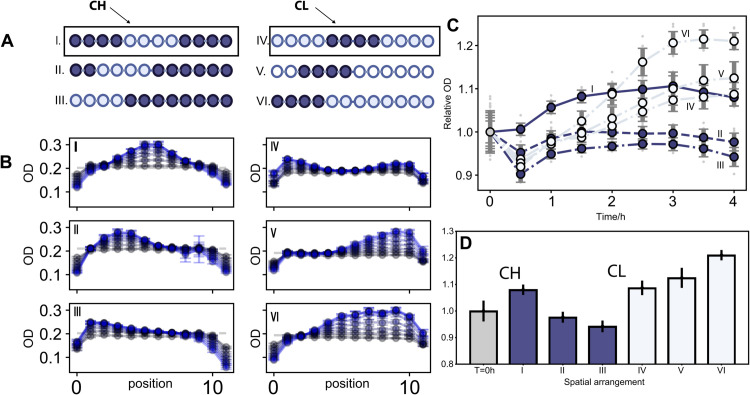
Different spatial arrangements lead to different temporal dynamics and collective responses. **A.** Six different spatial arrangements are depicted. Each column has the same number of wells with drugs, resulting in the same spatially averaged growth rate. Dark color indicates drug wells and light color indicates drug-free wells. The first column contains 8 wells with drugs; the central region initially has 4 drug-free wells with high growth rates, named Central High (CH). The second column contains 4 wells with drugs, and the central region initially has 4 high-drug wells with low growth rates, named Central Low (CL). In both CH and CL configurations, the central region is shifted two wells to the left in each subsequent arrangement (II,III) and (V,VI). **B.** The temporal dynamics of the six different spatial arrangements, with error bars corresponding to ±1 standard deviation of 8 technical replicates. Dashed line represents the initial OD at *T* = 0. Grey blue dots and curves are early cycles while light blues are late cycles. **C** and **D** present a comparison of the averaged temporal dynamics and final outcomes across six different spatial arrangements. In both panels, the y-axes represent the relative final ODs, normalized by dividing by the initial OD for comparison purposes, averaged over space and 8 technical replicates, with error bars corresponding to standard deviation. Dark blue represents I,II,III, while light blue represents IV,V,VI.

To begin, we designed six example spatial drug arrangements (Fig 3A, I–VI):

1. center drug-free wells (I),
2. left-side drug-free wells (II),
3. left-edge drug-free wells (III),
4. center drug wells (IV),
5. left-side drug wells (V),
6. left-edge drug wells (VI).

Center drug-free wells are denoted CH, as the highest growth rates occur at the center; similarly, CL denotes configurations with center drug wells. Configurations I–III all have *n*_*D*_ = 8, while IV–VI have *n*_*D*_ = 4. For each group, we examine how growth dynamics are influenced by spatial arrangement and compare responses within and across groups. Fig 3B shows the temporal dynamics of the six examples, illustrating reshaped density trajectories as expected from the spatial drug arrangements. A clear pattern emerges within both groups: as drug-free wells are positioned closer to the center of the spatial domain, final optical densities (ODs) become higher (I>II>III and IV<V<VI; see Fig 3C and 3D). Populations in II and III decline, whereas populations in I, IV, V, and VI grow. This provides direct experimental evidence of spatial arrangement effects. Both growth and decline occur with the same number of drug wells, unlike what’s predicted in the phase diagram in Fig 2C by the classical boundary condition $\lambda_{0}=\langle g\rangle \;-\;\frac{\pi^{2}\beta }{L^{2}}=0$. This suggests that, in the ⟨g⟩–$\frac{\beta }{L^{2}}$ phase diagram, under certain conditions, a new “arrangement-dependence” phase of mixed response may emerge, modulated by different spatial drug heterogeneities.

Although I–III have lower averaged growth rates and might be expected to yield lower final ODs than IV–VI (which have half as many drug wells), our results show that the center drug-free arrangement (I) produces final ODs almost as high as those of the center drug arrangement (IV) or the edge drug-free arrangement (III). In Fig K, panel A in S1 Text, a repeated experiment even shows that population I grows while population IV declines, indicating an even stronger advantage of CH over CL. This discrepancy between separate experiments may result from day-to-day fluctuations in drug concentration or temperature. Nevertheless, both datasets support the hypothesis that arrangements with the highest growth rates at the center maximize population growth, while those with the lowest growth rates at the center maximize population decline. Our simulation results (Fig K in S1 Text) closely match the observed temporal dynamics and population outcomes.

Together, these results show that population responses depend sensitively on spatial drug arrangements, and cannot be explained solely by the homogeneous $\langle g\rangle \;-\;\frac{\beta }{L^{2}}$ phase diagram (Fig 2C). Instead, they point to the existence of a new “mixed” phase determined by spatial drug arrangements, in which either growth or decline can occur. Moreover, the CH and CL arrangements may serve as upper and lower bounds for this mixed phase. These findings motivate further investigation of the growth–migration ($\langle g\rangle -\frac{\beta }{L^{2}}$) phase diagram to establish the generality of these spatial effects.

### Theory validates the indicated “arrangement-dependent” phase of mixed responses, and explains optimal spatial arrangements

**Fig 4:**
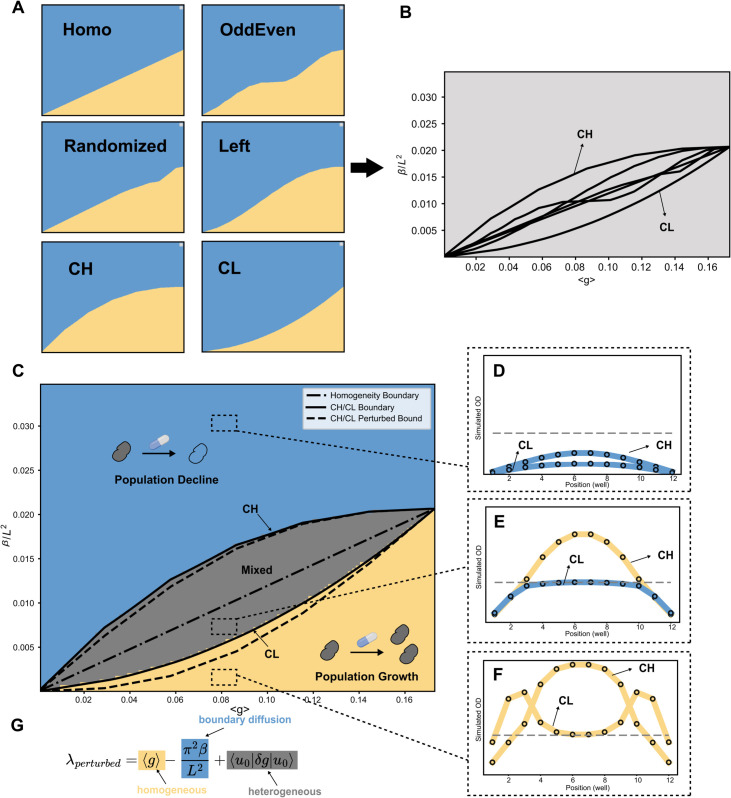
Model validation of the mixed response phase, optimal spatial arrangements CH and CL. **A**. numerical results of six different example spatial arrangement strategies. Blue phase represents population decline while orange color represents population growth. **B**. A comparison of six spatial arrangements by separatrices. Separatrices of CH and CL envelope all the other separatrices, implying that CH and CL can possibly be separatrices of the emerging mixed phase in the phase diagram of ⟨g⟩ and $\frac{\beta }{L^{2}}$. **C.** Phase diagram with new mixed phase by numerically solving the constrained optimization problem Eq 5. The solid lines are CH(upper) and CL(lower). They match with the numerical boundary well. The dash-dotted line is homogeneous spatial arrangement. Dotted lines are CH(upper) and CL(lower) by perturbation theory. **D,E,F.** 3 examples are taken from decline, mixed, growth phase. ⟨g⟩=0.0865, and $\frac{\beta }{L^{2}}=0.030,0.006,0.0012$. **G.** A decomposition of spatial drug homogeneous effect, boundary diffusion effect, and spatial drug heterogeneous effect of largest eigenvalue by perturbation approximation. It indicates that the diverging responses are incurred roughly by $\left\langle u_{0}|\delta g|u_{0}\right\rangle$, an average of spatial growth deviations weighted by square of unperturbed eigenvectors. The clip-art icon of the drug capsule was created in BioRender. Hu, Z. (2025) https://BioRender.com/zq812ma.

For a fixed spatially averaged growth rate ⟨g⟩, we can design different strategies for assigning growth rates across wells between 0 and the drug-free growth rate *g*_0_, while keeping ⟨g⟩ constrained. To begin, we implemented six spatial arrangement strategies for comparison: Homo, OddEven, Randomized, Left, CH, and CL. As indicated by our spatial arrangement experiments, each fixed arrangement strategy produces a distinct phase diagram in the ⟨g⟩–$\frac{\beta }{L^{2}}$ plane (Fig 4A). The “Homo” strategy distributes the averaged growth rate ⟨g⟩ evenly across all wells, recovering the phase boundary shown in Fig 2C. The “OddEven” strategy - assigning growth rates to odd-numbered wells first until saturation at *g*_0_, then to even wells - yields a more curved growth–decline boundary. The “Randomized” strategy assigns growth rates randomly to wells and produces a phase diagram nearly identical to homogeneity. The “Left” strategy, which sequentially assigns growth rates from left to right, produces a visibly different pattern. The “CH” strategy places higher growth rates at center wells first, whereas “CL” places them at edge wells first. Comparing all six strategies, CH results in the largest region of population growth, while CL yields the largest region of population decline, serving as bounds on all six strategies (Fig 4A and 4B).

To validate the hypothesis that the CH and CL arrangements maximize population growth and decline, and thus act as the upper and lower separatrices of the “arrangement-dependent” mixed phase, we formulate a constrained optimization problem:

$$\min_{\{g_{i}{\}}_{i=1}^{L}}\lambda_{0}=\|\Omega \|,\;\max_{\{g_{i}{\}}_{i=1}^{L}}\lambda_{0}=\|\Omega \|,\;\text{s.t. }\langle g\rangle =C,\;\;0\leq g_{i}\leq g_{0}.$$ (5)

Here, the equation is discretized for *L* = 12 wells, matching our experimental setup. The optimization is constrained by a fixed spatially averaged growth rate ⟨g⟩, while each well’s growth rate is restricted to the interval [0, *g*_0_] according to drug concentration. The minimizer of $\lambda_{0}=0$ corresponds to the lower separatrix of the mixed phase, while the maximizer that yields $\lambda_{0}=0$ corresponds to the upper separatrix. When the largest eigenvalue is positive for all spatial arrangements, the population always grows; when it is negative for all arrangements, the population always declines (Fig 4C). The mixed phase contains cases in which the outcome depends on spatial arrangements, and it is bounded by CH and CL, as proven using the KKT conditions (Sect 1.4 in S1 Text). Thus, CH and CL respectively mitigate population decline most and least effectively, corresponding to the largest growth and decline regions in the growth–migration phase diagram. Fig 4D–4F show examples of the decline phase, mixed phase, and growth phase, where (D) both CH and CL decline, (E) CH grows while CL declines, and (F) both grow. This ⟨g⟩–$\frac{\beta }{L^{2}}$ phase diagram demonstrates the existence of a mixed-response phase determined by spatial arrangements under certain parameter conditions, and indicates that even under spatial drug heterogeneity, bacterial population decline can be robustly guaranteed, by driving the system into the decline region of the diagram (Fig 4C).

To further understand this phase diagram, and to explain why the mixed phase is symmetric around the homogeneous growth-rate boundary, we apply first-order perturbation theory. The largest eigenvalue can be decomposed into three contributions: the homogeneous growth rate ⟨g⟩, the boundary diffusion term $\frac{\beta }{L^{2}}$, and the heterogeneous contribution $\left\langle u_{0}|\delta g|u_{0}\right\rangle$ induced by spatial drug arrangement (Fig 4G). The wells can be ranked by the squared components of the eigenvector $u_{0}(i{)}^{2}$. Given $u_{0}(x{)}^{2}=\frac{2}{L}\sin^{2}\;\left(\frac{(k+1)\pi x}{L}\right)$ (or $u_{0}(i{)}^{2}=\frac{2}{L+1}\sin^{2}\;\left(\frac{i\pi }{L+1}\right)$ in the discrete form), the center wells contribute the most weight (see Fig A in S1 Text). Consequently, placing drug-free high-growth wells at the center (CH) maximizes $\lambda_{0}$, as expected. Thus, the optimal spatial arrangements can be approximated by the shape of the leading eigenvector determined solely by boundary diffusion. The perturbative approximation is most accurate when boundary diffusion dominates (Fig B in S1 Text).

### Experimental data charts the new mixed phase and empirical separatrices

**Fig 5:**
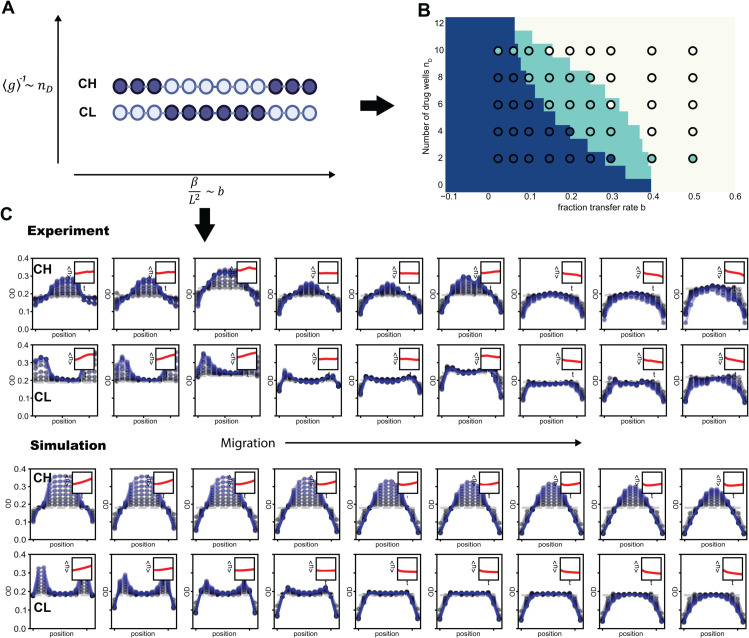
Experimental validation of the new mixed phase. **A.** Increase the number of drug wells *n*_*D*_ to decrease the spatially averaged growth rate ⟨g⟩, and increase the fraction of volume transfer *b* to enhance the boundary diffusion effect $\frac{\beta }{L^{2}}$. The example shown is *n*_*D*_ = 6(dark color indicates drug wells). **B.** Experimental data (dots) reveal three distinct regimes across the parameter space defined by the number of drug wells *n*_*D*_ and the fraction transfer rate *b*. The background shading represents the phases predicted by the model: population growth (dark blue, left side), mixed (light green, middle), and population decline (light yellow, right side). Experimental outcomes qualitatively match the model phases, confirming the emergence of the mixed phase. Another independent experimental phase diagram based only on endpoint data also confirms this finding (see Fig L in S1 Text). **C.** For the number of drug wells *n*_*D*_ = 6, by increasing the migration rate/fraction of volume transfer *b*, the population responses of both CH and CL shift from growing to declining (for each row, values of *b* correspond to panel **B**, and from left to right we have *b* = {0.025, 0.0625, 0.1, 0.15, 0.225, 0.25, 0.3, 0.4, 0.5}). The simulation captures the experimental features of these responses and their temporal dynamics. The population under the CH spatial arrangement continues to grow until *b* = 0.3, while the population under the CL arrangement grows only until *b* = 0.1. The inset shows the change in spatially averaged population density, ⟨u⟩, over time. The dashed line represents the initial optical density (OD). Grey blue dots and curves correspond to early cycles, while lighter shades of blue indicate later cycles. Each curve is the average of 8 technical replicates and the error bars correspond to standard deviation.

To validate the theoretical finding that a new mixed phase exists—where different spatial arrangements produce distinct dynamic outcomes in addition to the classical decline and growth phases—we experimentally tested various fraction transfer rates *b* and numbers of drug wells *n*_*D*_. These correspond to tuning the migration rate *β* and the spatially averaged growth rate ⟨g⟩ (Fig 5A and 5B). To avoid the curse of dimensionality associated with all possible permutations, we focused on the center drug-free (CH) and center drug (CL) arrangements from the new phase diagram, as they define the largest region of the mixed phase and therefore yield the most distinct results (Fig 4C). As described in the system setup, whether the population declines or grows is determined by the sign of $\lambda_{0}$.

For a fixed number of drug wells (e.g., *n*_*D*_ = 6; Fig 5C), we experimentally increased the fraction of transferred volume *b* to strengthen the boundary diffusion effect. We found that both CH and CL populations grow when the migration rate (boundary diffusion effect) is small. As the boundary diffusion effect increases, the population under the CL arrangement begins to decline, whereas the CH arrangement continues to support growth. Eventually, when the boundary diffusion effect becomes sufficiently large, both populations decline. Our simulations qualitatively reproduce these temporal dynamic features (Fig 5C; see also Figs E-J in S1 Text).

The experimental endpoint outcomes (dots) agree well with the phase diagram obtained by numerically solving the eigenvalues for the CH and CL arrangements (Fig 5B). The mixed phase remains visible in the intermediate region, where CH grows while CL declines. In Fig L in S1 Text, we provide an additional independently repeated experimental phase diagram. For simplicity, only endpoint densities were measured in that experiment. Although the repeated dataset contains fewer points within the mixed phase (likely due to day-to-day fluctuations in drug concentration and temperature), it still supports the existence of the mixed phase identified in our theory and primary experiments.

### Spatial effect in other spatially-extended systems beyond the classic framework

In the previous sections, we demonstrated how the interplay between spatial drug heterogeneity and boundary diffusion can lead to distinct population-level responses. In our earlier case, spatial heterogeneity was generated using a bacteriostatic drug, producing a non-negative growth landscape across space. Here, population loss arose solely from boundary diffusion, allowing us to isolate the role of spatial heterogeneity itself. However, killing can also arise directly from spatial heterogeneity when bactericidal drugs are used. Unlike bacteriostatic agents, bactericidal drugs actively kill bacteria [32]. At sufficiently high concentrations, the killing rate can exceed the intrinsic growth rate, resulting in a net negative growth rate. In such a source–sink configuration, some wells (sources) with low drug concentrations sustain positive growth, while others (sinks) with high drug concentrations impose negative growth. Migration from sources to sinks causes a fraction of the population to die upon arrival [14,50], driving overall population decline. In this scenario, growth, migration, and killing are intertwined, complicating direct analysis.

To provide a minimal yet illustrative example, we extend our mixed-phase findings to a source–sink setting using a ring-shaped spatial structure with periodic boundaries. This geometry removes the boundary diffusion effect entirely and thus lies beyond the classic “critical-patch-size” framework. While periodic boundary conditions have been widely studied in theoretical ecology across finite and infinite one- or two-dimensional domains [37,51–54], to our knowledge, the mixed phase has received little attention, and experimental evidence remains scarce. As a minimal experimental analogue, we applied the bactericidal drug ampicillin, commonly used clinically to eradicate *E. faecalis*, to specific wells to induce maximal cell lysis [32], thereby creating sinks, while drug-free wells served as sources. The interplay between the maximal death rate in sinks, the growth rate in sources, and the migration rate *β* determines whether the population persists or collapses.

**Fig 6:**
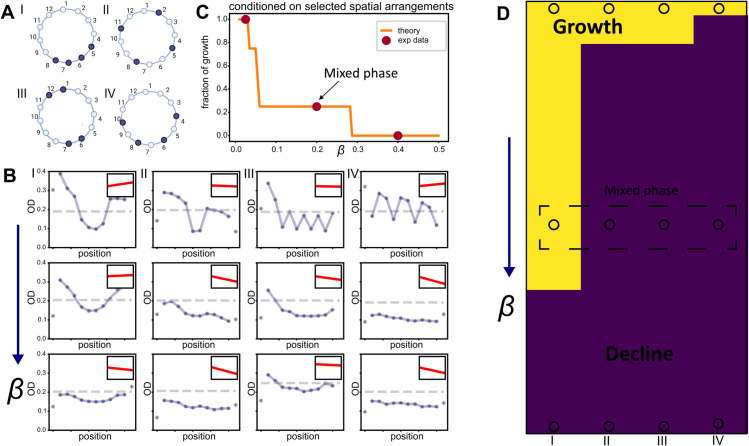
Different spatial drug arrangements on a ring structure induce divergent response outcomes. **A.** Four different spatial arrangements (I, II, III, IV) in a ring structure, where dark color indicates drug wells (*g* < 0), and light color indicates drug-free wells (*g* > 0). **B.** These four spatial arrangements orchestrate different temporal dynamics across three different migration regimes. Dashed line represents the inital OD while blue dots and curves represent endpoint ODs. The inset shows the change in spatially averaged population density with 8 technical replicates, ⟨u⟩, over time. **C.** Among the four spatial arrangements, the fraction of growth responses decreases as the migration rate increases; at β=0.2, the fraction is 0.25, indicating the existence of a mixed phase depending on spatial arrangements. **D.** The experimental data (dots) aligns with the numerical phase diagram obtained by solving the largest eigenvalue. Yellow indicates growth responses, while dark blue indicates decline responses.

It is intuitive that at low migration rates, bacteria thrive in drug-free wells with minimal perturbation from the high-drug sinks [55]. As the migration rate *β* increases, populations under different spatial arrangements diverge into a mixed phase and eventually decline at high migration rates, mirroring the behavior in the phase diagram with boundary diffusion (Fig 4C). Our experimental results across four spatial arrangements confirm this: as *β* increases, the fraction of population growth (number of arrangements showing growth over the total) transitions from 1 (growth phase), to 0.25 (mixed phase), and finally to 0 (decline phase) (Fig 6C). Thus, despite differences in drug type and boundary conditions, the migration rate remains a key driver of population decline, and the system exhibits similar diverging outcomes.

We next examined whether our reaction–diffusion model with periodic boundary conditions and explicit death rates qualitatively aligns with the experimental data. Fig 6B shows temporal dynamics across migration rates and spatial drug arrangements averaged by eight technical replicates, closely matching simulations (Fig M in S1 Text). Theoretical predictions using the largest eigenvalue criterion also accurately capture the observed outcomes (Fig 6D). These agreements validate the robustness of our framework in more complex spatial settings.

To explain the emergence of spatial arrangement effects in this ring structure, we computed the perturbed eigenvalue. However, because all wells are equivalent in this geometry, the perturbed largest eigenvalue becomes $\lambda_{p}=\langle g\rangle$, equal to the spatially averaged growth rate. As a result, it no longer reflects spatial drug heterogeneity and cannot decompose the contributions of growth, migration, and killing. This suggests that higher-order perturbative methods or new theoretical tools may be required to analyze more complex spatial configurations.

## Discussion and conclusion

In this paper, we developed a minimal experimental system automated by a pipetting robot, to investigate the effects of spatial drug heterogeneity on a confined 1D inhabitat. We first recapitulated the classic “critical-patch-size” model result, and then found out that, different spatial arrangements of drugs, even with the same spatially averaged growth rates, can lead to divergent bacterial population outcomes, resulting in a “arrangement-dependent” phase of mixed responses, or “mixed” phase. Furthermore, simulation and optimization identify CH and CL (CH refers to the center drug-free wells, as the highest growth rates occur at the center; CL denotes configurations with center drug wells) as two optimal spatial arrangements, serving as empirical upper and lower bounds of this mixed phase. This finding is validated through systematic high-throughput experiments with different parameter sweepings. A perturbation-theory approximation further explains how spatial drug arrangements alter growth dynamics and lead to different outcomes. It also illustrates that even under spatial drug heterogeneity, it is possible to ensure bacterial population decline regardless of spatial or temporal drug fluctuations by driving the system from the mixed phase into the decline phase. Extensions using a ring structure confirm the existence of such “arrangement-dependent” effect, demonstrating that spatial heterogeneities can generate diverging population responses in spatially extended systems beyond the classic KiSS framework.

The one-dimensional Fisher–KPP equation, $\frac{\partial u}{\partial t}=\beta \frac{\partial^{2}u}{\partial x^{2}}\;+\;f(u,x,t),$ is a well-known model in ecological and evolutionary dynamics, describing cell growth and range expansion in spatially varying environments [56,57]. Here, we consider a linearized form with fully absorbing boundary conditions known as KiSS model [35,36]. In our model, the largest eigenvalue separates into two components describing the effective growth ($g_{eff}$) and the boundary diffusion effect ($\beta /L^{2}$). The population declines when $\lambda_{0}=g_{eff}\;-\;\frac{\pi^{2}\beta }{L^{2}}<0.$ For homogeneous environments, $g_{eff}=\langle g\rangle =g(D)$ is the growth rate under uniform drug concentration. For heterogeneous environments, $g_{eff}$ can be approximated using first-order perturbation theory as $g_{eff}=\langle g\rangle +\langle u_{0}|\delta g|u_{0}\rangle .$ If $\beta /L^{2}\gg \langle g(x)\rangle$, populations decline; if $\beta /L^{2}\ll \langle g(x)\rangle$, populations persist and grow despite the deleterious environment. Under spatial heterogeneity, the classical critical boundary becomes a “mixed phase”. Under periodic boundaries with bactericidal drugs such as ampicillin, the condition becomes u(0,t)=u(L,t), and population loss arises from sink wells with negative growth rates rather than boundary diffusion.

The concept of a critical patch size is foundational in spatial ecology and reaction–diffusion theory, showing that below a threshold habitat size, populations cannot persist [39–42,58]. Prior work mostly examined homogeneous environments or idealized growth conditions. Recent studies on antibiotic resistance under spatial drug gradients further explored these ideas, but did not explicitly isolate how spatial heterogeneity in drug-induced growth rates, combined with patch size and migration, affects survival. Here, we provide the first experimental demonstration that the spatial arrangement of antibiotics, even at fixed average drug levels, can dramatically alter bacterial outcomes in confined environments.

For the two optimal arrangements (CH and CL) with fixed average growth rates, their opposing yet symmetric structures arise from the effects of boundary diffusion and the symmetry of the 1D system. Similar “positional advantages” have been observed experimentally, such as in evolution experiments conducted in microchannels with absorbing boundaries [59], where central positions exhibit dominance advantages. This highlights the critical role of boundary conditions. Beyond bacterial systems, spatial structures—and potentially more general network topologies—play important roles in cancer therapies and clinical decisions, often manifesting as star or tree-like configurations [60,61]. Since a mixed phase still emerges in these settings, further research is required to explore how spatial structure influences optimal drug arrangements.

To avoid potential mutations during long-term experiments and maintain stable environmental conditions, we omitted the dilution step commonly used in range-expansion experiments [56]. While efficient, this approach may allow limited drug diffusion; we minimized this effect by selecting appropriate experimental parameters (Sect 5 and Figs P-V in S1 Text). Drug diffusion, however, is likely to be significant in vivo, where pharmacokinetic–pharmacodynamic (PK–PD) processes operate over long timescales. Our experimental mixed-phase region appears narrower than the ideal theoretical prediction, likely reflecting this additional noise. Although our model is simplified relative to experimental complexity, it remains sufficiently powerful to qualitatively explain the observed phenomena. These discrepancies imply that drug diffusion and other time-varying fluctuations remain important considerations, and that clinical investigation of spatial drug heterogeneity should minimize environmental noise to clearly identify mixed-phase behavior.

Although this study focused on single-species WT dynamics under a single drug, natural microbial communities often involve multiple interacting species and multiple drugs administered together. Based on the same framework, our recent work has theoretically explored how antibiotic-resistant mutants are selected among multiple strains under spatial multi-drug heterogeneities [54]. More experimental and clinical data are needed to validate these findings. Moreover, while our system assumes density-independent exponential growth [28,62], ecological interactions within populations and among species can significantly modify outcomes [63–70]. Such interactions may lead to counterintuitive behavior [67,68] and contribute to increased antibiotic resistance [63]. Understanding how spatial drug heterogeneity influences these ecological processes remains an open question. Recent work also emphasizes diversity-dependent dispersal, where interspecies interactions shape spatial dynamics [71,72]. Metapopulation models further suggest that quenched disorder in death rates can induce novel coexistence phases in systems with migration and species interactions [73], offering promising directions for future research.

Personalized therapies have gained increasing attention, as identical treatments can lead to diverging clinical outcomes [74,75]. Bistable population outcomes driven by density effects have been observed in previous *E. faecalis* studies [76]. The mixed phase described here provides an additional potential explanation: patients with identical drug doses may experience different therapeutic outcomes due to differing spatial drug distributions in their tissues, which may determine whether pathogens are cleared.

To summarize, by linking theory and experiments, we have shown that in a deleterious confined environment where growth rates are spatially modulated by drug heterogeneity, ecological dynamics and population responses depend strongly on spatial arrangement — not merely on the total drug amount. This highlights the importance of understanding and potentially tuning spatial drug distributions in clinical contexts, especially given the growing challenges of pathogen persistence and cancer metastasis under treatment.

## Supporting information

S1 Text The Supplementary S1 Text contains expanded description of mathematical models (Section “1D simplified Fisher-KPP model in a deleterious confined environment”), details of experimental design and parameter estimation (Section “Experimental-related design and data analysis”), simulations and repeated experiments (Section “Simulations and repeated experiments support spatial drug arrangement effect”), discussions on largest eigenvalue and long-term growth rate, drug diffusion effect, active migration with a metabolic cost, gradual concentration change over space (the rest sections), and 22 supplemental figures. (PDF)
