# Supplementary material for: Linking spatial drug heterogeneity to microbial growth dynamics in theory and experiment
Source: PLoS Comput Biol. 2026 Jan 20;22(1):e1013896. doi: 10.1371/journal.pcbi.1013896 (PMC12863682; doi:10.1371/journal.pcbi.1013896)
Supplement: S1 Text — (PDF) [file pcbi.1013896.s001.pdf]

S1 Text: "Linking spatial drug heterogeneity to microbial growth dynamics in theory and experiment"

1 1D simplified Fisher-KPP model in a deleterious confined environment

Species invade new territory through a combination of dispersal and local growth. Migration or diffusion in a free environment, like range expansions, are well captured by a simple model incorporating growth and nearest-neighbor dispersal, known as Fisher–Kolmogorov–Petrovsky–Piskunov equation, or Fisher-KPP equation[1, 2],

$$\frac{\partial u}{\partial t} = \beta \frac{\partial^2 u}{\partial x^2} + g(u)u \tag{S1}$$

where β is the diffusion or migration rate, u is the cell density, and $g(u)$ is the density-dependent growth rate. To describe the bacteria migration in a confined environment with deleterious boundaries, mathematically, 2 absorbing boundaries are attached and growth rate are linearized for simplicity, by ignoring the intra-species competition

$$\begin{cases} \frac{\partial u}{\partial t} = \beta \frac{\partial^2 u}{\partial x^2} + g(D(x))u \\ u(x,0) = u_0 \\ u(0,t) = 0 \\ u(L,t) = 0 \end{cases} \tag{S2}$$

Now bacteria population are confined within a system of size L. $g(D(x))$ is the density-independent growth rate modulated by drug concentration D, distributed over x. u_0 is the initial cell density over space and we here assume it's uniform. To align our 1D experimental system featuring disjointed patches, this modified Fisher-KPP equation can be further discretized, thereby both mimicking metapopulation dynamics typical of many ecological and experimental systems, while also the continuous dynamics frequently assumed in mathematical ecology[1].

1.1 Transformation to an eigenvalue problem

The Fourier method (also known as separation of variables) is applied below to solve the reaction-diffusion equation. Readers can also try the other methods for their own interest. Consider the following form separating the solution into a product of spatial and temporal components

$$u(x,t) = u_x(x)u_t(t) \tag{S3}$$

Thus the PDE can be re-written as

$$u_x\dot{u}_t = \beta u_x'' u_t + g(D(x))u_x u_t \tag{S4}$$

where u_x'' refers to second-order spatial derivative of u_x. Divide both sides by $u_x u_t$ and let it equal to a constant λ,

$$\frac{\dot{u}_t}{u_t} = \beta\frac{u_x''}{u_x} + g(D(x)) = \lambda \tag{S5}$$

Rewrite it as a pair of ODEs with respect to u_t and u_x,

$$\frac{\partial u_t}{\partial t} = \lambda u_t, \tag{S6}$$

$$\beta\frac{\partial^2 u_x}{\partial x^2} + g(D(x))u_x = \lambda u_x \tag{S7}$$

where $x \in [0, L]$, $u_t(0) = u_0$, $u_x(0) = 0$ and $u_x(L) = 0$. The solution of the former is $u_t = u_0 e^{\lambda t}$ while the latter defines the eigenvalue problem,

$$\Omega u_k = \left(g(D(x)) + \beta\frac{\partial^2}{\partial x^2}\right)u_k = \lambda u_k. \tag{S8}$$

where Ω is the operator incorporating growth and migration, λ_k is the corresponding kth eigenvalue and $k = 0, 1, 2, \cdots, \infty$. combining the temporal and spatial solutions, the general solution $u(x,t)$ now has the form

$$u(x,t) = \sum_{k=0}^{\infty}\langle u_0|\psi_k\rangle e^{\lambda_k t} \tag{S9}$$

$\langle u_0|\psi_k\rangle = \int_0^L u_0(x)\psi_k(x)dx$ is the coefficient where the initial cell density projected to the eigenbasis. In the long time limit, the cell density are dominated by its largest eigenvalue $u(x,t) \approx \langle u_0|\psi_0\rangle e^{\lambda_0 t}$. When $\lambda_0 < 0$, the whole population goes down and eventually extinct. Thus λ_0 here can be a good metric to check whether population declines or not. For comparison with the experimental results, we can numerically solve the eigenvalue equation to get λ_0.

To rescale the spatial coordinate x by L, let $\tilde{x} = x/L$ we have

$$\Omega u_{\tilde{x}} = \left(g(D(\tilde{x}L)) + \frac{\beta}{L^2}\frac{\partial^2}{\partial\tilde{x}^2}\right)u_{\tilde{x}} = \lambda u_{\tilde{x}}. \tag{S10}$$

$\tilde{x} \in [0,1]$ and now we get our rescaled migration $\frac{\beta}{L^2}$. It incorporates the effect of migration rate β and system size L. Since in our experiments we either fix migration rate or system size, we won't distinguish rescaled migration or the original migration. For simplicity, let's write back the notation x

$$\Omega u_x = \left(g(D(x)) + \frac{\beta}{L^2} \frac{\partial^2}{\partial x^2} \right) u_x = \lambda u_x. \tag{S11}$$

1.2 Exact eigenvalue solution under spatial drug homogeneity

Assume that the drug concentration is homogeneous over space so $g(D(x)) = g(D) = \langle g \rangle$, the last term is spatially averaged growth rate; in this paper we use this notation to refer growth rate level under homogeneity. Then it becomes a well studied linear Sturm-Liouville system and has an exactly solvable solution because of its integrability. So

$$\lambda_k - \langle g \rangle = -\frac{\beta \pi^2 (k+1)^2}{L^2} \tag{S12}$$

And the corresponding eigenvector is $\psi_k = \sqrt{\frac{2}{L}} \sin\left(\frac{(k+1)\pi x}{L}\right)$.This shares the same form as the eigenvectors of pure diffusion equation $\beta \frac{\partial^2 u_x}{\partial x^2} = \lambda u_x$. The population decline criterion is given by the largest eigenvalue $\lambda_0 = \langle g \rangle - \frac{\pi^2 \beta}{L^2} < 0$. As mentioned in the main text, the first term is our homogeneous growth rate and the second term is the boundary diffusion effect. They together account for the interplay.

1.3 First-order perturbation approximation under spatial drug heterogeneity

Although for a general form of the growth rate $g(D(x))$ under spatial drug heterogeneity, the eigenvalue is not explicitly solvable, we aim to design an approximation that separates the forces driving population growth and decline—namely, the growth rate and boundary effects, as in the homogeneous case. A simple mean field approximation (zero-order approximation) is inadequate here, as it merely yields the spatial average $g_{MF} = \langle g \rangle$, thus neglecting information about spatial arrangement. Instead, we consider a first-order spatial effect using perturbation theory. In our recent work [3], this method demonstrated excellent agreement with numerical eigenvalues, even under multiple-drug resistance evolution with drug interactions and collateral effects. By rewriting the original eigenvalue equation, we can effectively separate the heterogeneous effects.

$$\beta \frac{\partial^2 u_\lambda}{\partial x^2} + \langle g \rangle u_\lambda + \delta g u_\lambda = \lambda u_\lambda \tag{S13}$$

$\delta g = g(D(x)) - \langle g \rangle$ describes the heterogeneous growth deviation and $\langle \delta g \rangle = 0$, modulated by spatial drug heterogeneity. Choose growth with homogeneous drug concentration as the unperturbed system, so we have $u_k = \psi_k = \sqrt{\frac{2}{L}} \sin\left(\frac{(k+1)\pi x}{L}\right)$ as our eigenbasis. The largest eigenvalue λ_0 now becomes[3]

$$\lambda_0 \approx \lambda_{perturbed} = \langle g \rangle - \frac{\pi^2 \beta}{L^2} + \langle u_0 | \delta g | u_0 \rangle \tag{S14}$$

u_0 is the unperturbed eigenvector corresponding to the largest eigenvalue, from the original unperturbed system. This holds when the homogeneous growth rate $\langle g \rangle$, or migration rate β, is much larger than the heterogeneous growth deviation δg. So under the high migration or small spatial fluctuation regime, as shown in Figure 5A in the main text, our approximation has small relative errors. It's general for any spatial drug arrangements. We can also elaborate it in another way. Observe that the exact solution $\lambda_0 = \langle u_p | \Omega | u_p \rangle = \langle g \rangle + \langle u_p | \beta \frac{\partial^2 u_\lambda}{\partial x^2} | u_p \rangle + \langle u_p | \delta g | u_p \rangle$, since $\langle u_p | u_p \rangle = 1$. When the perturbed eigenvector u_p close to the unperturbed eigenvector u_0, we recover $\lambda_0 \approx \langle g \rangle - \frac{\pi^2 \beta}{L^2} + \langle u_0 | \delta g | u_0 \rangle = \lambda_{perturbed}$. It's illustrated by Figure 5C, showing that a fraction of u_p are close to the unperturbed u_0, of all the possible u_ps from the boundaries of mixed phase.

As stated in main text, the optimal spatial drug arrangement, CH or CL, is explainable by perturbation theory. To optimize the largest eigenvalue, we need to optimize $\langle u_0 | g(x) | u_0 \rangle$. Rewrite $\langle u_0 | g(x) | u_0 \rangle = \frac{2}{L} \int_0^L g(x) \sin^2\left(\frac{\pi x}{L}\right) dx$. So this is a weighted average of $g(x)$ by $\sin\left(\frac{\pi x}{L}\right)$. Noticing that spatially averaged growth rate is fixed, and we can derive the position x of its maximum value by setting $\frac{d \sin^2\left(\frac{\pi x}{L}\right)}{dx} = 0$, which gives $x = \frac{L}{2}$. So $\sin^2\left(\frac{\pi x}{L}\right)$ has the maximum value at center ($x = \frac{L}{2}$) and minimum value at boundaries ($x = 0, x = L$) according to its function form (see Figure A). Thus the maximized $\lambda_{perturbed}$ is acquired when we saturate the growth rate at the center as CH strategy. When we saturate the growth rate at boundaries as CL, we get the minimized $\lambda_{perturbed}$. This also holds in a discrete version of our eigenvectors $u_0(i)^2 = \frac{2}{L+1} \sin^2\left(\frac{i\pi}{L+1}\right)$.

1.4 Boundaries of mixed phase by constrained optimization

For simplicity, a discrete version of simplified Fisher-KPP model is chosen and let $L = 12$wells. To get the boundaries of mixed phase, we can solve the following constrained optimization problems

$$\begin{aligned}
&\min_{\{g_i\}_{i=1}^L} \; \lambda_0 = \|\Omega\| \\
&\max_{\{g_i\}_{i=1}^L} \; \lambda_0 = \|\Omega\| \\
&\text{s.t. } \langle g \rangle = C, \\
&\qquad 0 \leqslant g_i \leqslant g_0
\end{aligned} \tag{S15}$$

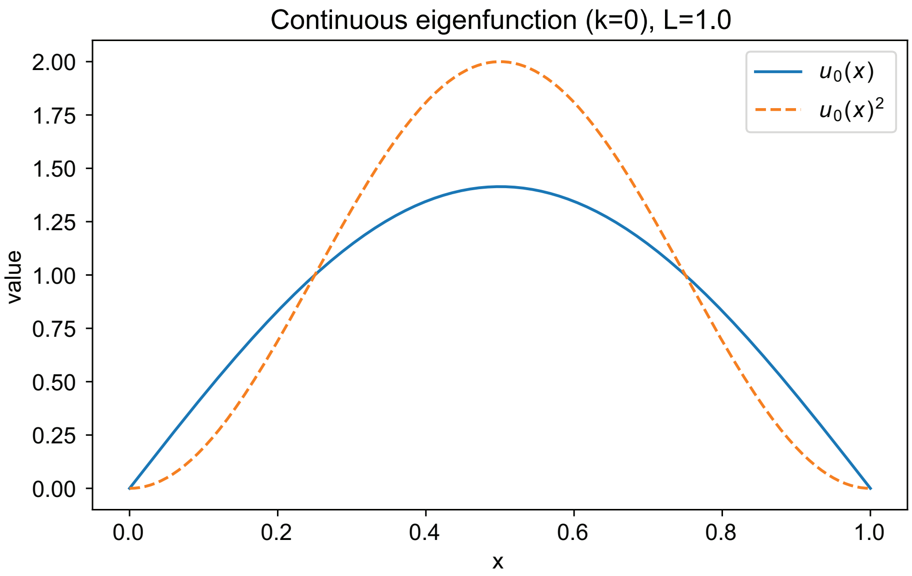

Figure A: **Plotting of $u_0(x)$ and $u_0(x)^2$ (k=0) in $[0, L]$, with L=1.0.** The maximum value is at center ($x = \frac{L}{2} = 0.5$) and minimum values are at boundaries ($x = 0, x = L = 1.0$).

where $\Omega = \beta\mathcal{L} + G$, $\mathcal{L} = \begin{bmatrix} -2 & 1 & & & \\ 1 & -2 & 1 & & \\ & \ddots & \ddots & \ddots & \\ & & 1 & -2 & 1 \\ & & & 1 & -2 \end{bmatrix}$ is the $L \times L$ matrix describing the migration between wells, or discretized Laplacian operator, $G = \begin{bmatrix} \ddots & & \\ & g_i & \\ & & \ddots \end{bmatrix}$ is the growth matrix with ith diagonal entry representing the growth rate at the ith well. The diagonal elements of G are tuned to get optimized largest eigenvalue. C is a constant value. The population growth, mixed, population decline phase are determined when $\lambda_{0,min} > 0, \lambda_{0,max} > 0$, $\lambda_{0,min} < 0, \lambda_{0,max} > 0$, $\lambda_{0,min} < 0, \lambda_{0,max} < 0$. With $\lambda_{0,min} = 0$ the rescaled migration β/L^2 is solved to determine the lower boundary of mixed phase. Solving β/L^2 from $\lambda_{0,max} = 0$ gives us the upper boundary of mixed phase.

To prove that CH, CL are the upper and lower boundaries of mixed phase, KKT condition is applied. Here we use the maximization problem as an example. For incorporating inequality constraints, we define the Lagrangian with

KKT multipliers μ_i and ν_i:

$$\mathcal{L}(\lambda_0, g_1, g_2, \ldots, g_L, \mu, \{\mu_i\}, \{\nu_i\}) = -\lambda_0 + \mu\left(\frac{1}{L}\sum_{i=1}^{L} g_i - C\right) + \sum_{i=1}^{L}\mu_i(g_i - g_0) - \sum_{i=1}^{L}\nu_i g_i \tag{S16}$$

The KKT conditions for this problem are:

1. **Stationarity**:

$$\frac{\partial \mathcal{L}}{\partial g_i} = -\frac{\partial \lambda_0}{\partial g_i} + \frac{\mu}{L} + \mu_i - \nu_i = -u(i)^2 + \frac{\mu}{L} + \mu_i - \nu_i = 0$$

2. **Primal Feasibility**:

$$\frac{1}{L}\sum_{i=1}^{L} g_i = C$$

$$0 \leqslant g_i \leqslant g_0 \quad \text{for all } i$$

3. **Dual Feasibility**:

$$\mu_i \geqslant 0, \quad \nu_i \geqslant 0 \quad \text{for all } i$$

4. **Complementary Slackness**:

$$\mu_i(g_i - g_0) = 0, \quad \nu_i g_i = 0 \quad \text{for all } i$$

The second equality from stationarity is derived by Hellmann–Feynman theorem,

$$\frac{\partial \lambda}{\partial g_i} = u^T \frac{\partial \Omega}{\partial g_i} u = u^T e_i e_i^T u = \left(u^T e_i\right)^2 = u(i)^2 \tag{S17}$$

From stationarity we can see that if none of the inequality constraints are active, $\mu_i = \nu_i = 0$, we have $\frac{\mu}{L} = u(i)^2$, for all positions. Since $u(i)^2$ are not uniform due to the boundary condition and spatial drug heterogeneity, so at least we have 1 active inequality constraint. And this gives

$$\begin{aligned} u(i)(g)^2 - \frac{\mu}{L} - \mu_i = 0 \qquad &\text{with} \quad \mu_i > 0, \quad g_i = g_0 \\ u(i)(g)^2 - \frac{\mu}{L} + \nu_i = 0 \qquad &\text{with} \quad \nu_i > 0, \quad g_i = 0 \\ u(i)(g)^2 - \frac{\mu}{L} = 0 \qquad &\text{with} \quad 0 < g_i < g_0 \end{aligned} \tag{S18}$$

And this indicates

$$u(i)^2 > \frac{\mu}{L}, \quad u(j)^2 < \frac{\mu}{L}, \quad u(k)^2 = \frac{\mu}{L} \tag{S19}$$

i, j, k are indices corresponding to drug-free wells, drug wells with full inhibition, and the critical well where the wells are transiting from drug-free wells to drug wells. So to maximize the eigenvalue, we assign g_0 at high $u(i)^2$, 0 at low

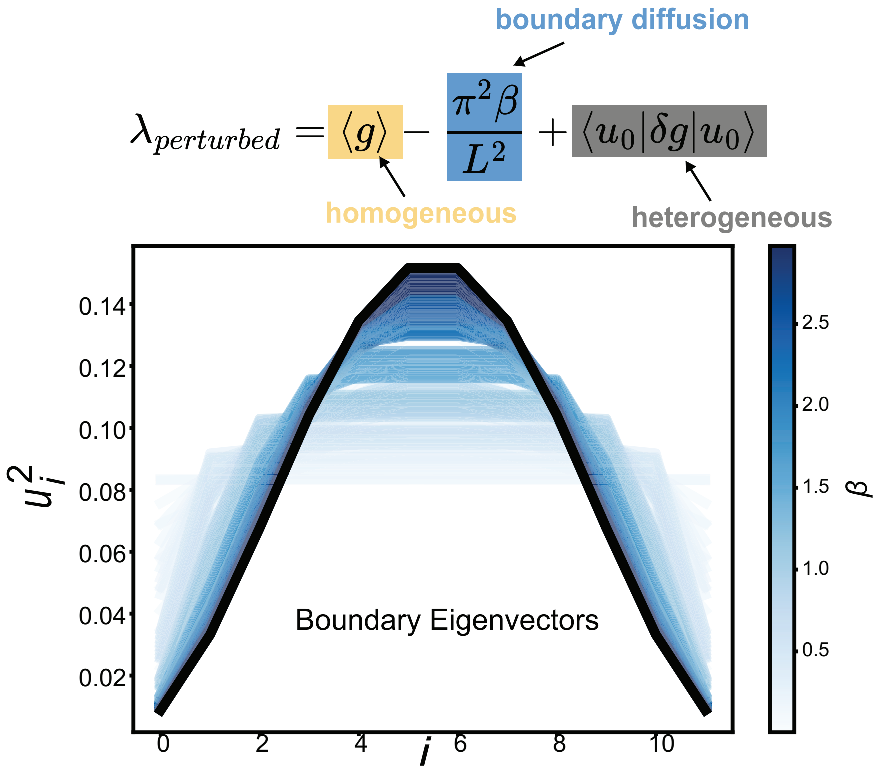

Figure B: **Unperturbed boundary eigenvectors $u_0(i)$ comparing with eigenvectors $u_0(i)$ under CH/CL spatial arrangements, in a squared form.** As migration rate β increases, all eigenvectors from CH and CL spatial arrangements with different spatially averaged growth rates $\langle g \rangle$, are getting closer to $u_0(i)$.

$u(i)^2$. The critical k is when $u(k)^2 = \mu/L$. Figure B shows that for $u(i)^2$ from the upper and lower boundaries, $u(i)^2$ are always highest at center and loweset at edge. Thus CH is the optimal spatial drug arrangement for maximized λ_0 and CL is optimal for minimized λ_0.

As rescaled migration rate increases, the perturbed eigenvector is closer to u_0. From the perturbed eigenvalue $\lambda_{perturbed} = \langle g\rangle - \frac{\pi^2\beta}{L^2} + \langle u_0|\delta g|u_0\rangle$, it's clear that we need to optimize $\langle u_0|\delta g|u_0\rangle = \sum u_0(i)^2(g_i - \langle g\rangle) = \sum \frac{2}{L+1}\sin^2\left(\frac{i\pi}{L+1}\right)(g_i - \langle g\rangle)$. Here we use a discrete version of u_0. So, still, because $u_0(i)^2 = \frac{2}{L+1}\sin^2\left(\frac{i\pi}{L+1}\right)$ is maximized at the center and minimized at the edge, we have CH give the maximizer and CL give the minimizer as upper and lower boundaries of the new mixed phase. The KKT condition gives that the optimal δg_i values lie at the bounds $-\langle g\rangle$ or $g_0 - \langle g\rangle$, depending on $u_0(i)^2$, and the average constraint ensures the solution is feasible.

Now we have everything to derive the boundaries $\frac{\beta}{L^2}(\langle g\rangle)$. Since $\lambda_0(\mathcal{L}) = -2\frac{\beta}{L^2}(1+\cos\left(\frac{\pi}{L+1}\right))$ in a discrete version, by solving $\lambda_0 = -2\frac{\beta}{L^2}(1+\cos\left(\frac{\pi}{L+1}\right)) + \sum_{i=1}^{L} u(i)^2 g_i = 0$ we get rescaled migration $\frac{\beta}{L^2} = \frac{\sum_{i=1}^{L} u(i)^2 g_i}{2L^2\left(1-\cos\left(\frac{\pi}{L+1}\right)\right)}$, with $\langle g\rangle = C$. This forms the boundary for any spatial arrangement including the homogeneity case, CH, and CL. From the perturbation theory we get

$$\frac{\beta}{L^2} \approx \frac{\sum_{i=1}^{L} u_0(i)^2 g_i}{L^2\left(1-\cos\left(\frac{\pi}{L+1}\right)\right)} = \frac{\sum_{i=1}^{L} \sin^2\left(\frac{i\pi}{L+1}\right) g_i}{L^2\left(1-\cos\left(\frac{\pi}{L+1}\right)\right)} \tag{S20}$$

1.5 Perturbed optimal phase boundaries are always lower

Rewrite the unperturbed and perturbed eigenvalue function

$$\beta\mathcal{L}\,|u_0\rangle = \lambda_0\,|u_0\rangle\,, \quad (\beta\mathcal{L} + G)\,|u\rangle = \lambda|u\rangle$$

The second equation can be written as $|u\rangle = \frac{1}{\lambda - \beta\mathcal{L}} G|n\rangle$. To eliminate the singularity at $G \to 0$, we define the projection operators

$$P_0 = |u_0\rangle\,\langle u_0| \quad \text{and} \quad Q_0 = 1 - P_0 = \sum_{u\neq q} |q_0\rangle\,\langle q_0|$$

Thus, we have $(1 - P_0)\,|u_0\rangle = Q_0\frac{1}{\lambda-\beta\mathcal{L}}G|u\rangle$. The right-hand side can be written as

$$gG|u\rangle \quad \text{where} \quad g = \sum_q \frac{|q\rangle\langle q|}{\lambda - \lambda_0}$$

Therefore, we obtain

$$|u\rangle = |u_0\rangle\,\langle u_0|u\rangle + gG|u\rangle$$

It's known as Lippmann-Schwinger equation.

It is obvious that this equation can be solved iteratively, and its form is

$$|u\rangle = \langle u_0|u\rangle\,(1 + gG + gGgG + \ldots)\,|u_0\rangle$$

$$\langle u|g|u\rangle = \langle u|g|\,\langle u_0|u\rangle\,(1 + gG + gGgG + \ldots)\,|u_0\rangle$$

However, we are interested in understanding eigenvalue perturbation rather than eigenstate perturbation. To solve this, we have

$$\langle u_0\,|\beta\mathcal{L} + G|\,u\rangle = \lambda\,\langle u_0|u\rangle$$
$$\text{LHS} = (\lambda_0 + \langle u_0|G + GgG + GgGgG + \ldots|u_0\rangle)\cdot\langle u|u_0\rangle = \text{RHS} = \lambda\cdot\langle u|u_0\rangle$$

Thus, we have

$$\lambda = \lambda_0 + \langle u_0|G + GgG + \ldots|u_0\rangle \quad \text{where} \quad g = \sum_{q\neq u}\frac{|q\rangle\langle q|}{\lambda - \lambda_0}$$

It seems that this is already the general solution, but since g contains the perturbed λ rather than λ_0, it still requires a series solution. However, it has been greatly simplified compared to before. Notice that for our specific system, i. G is a diagonal matrix, and ii.$\lambda_{perturbed} - \lambda_0 = \langle u_0|G|u_0\rangle > 0$, iii. $u_i^2 > 0$. So apparently $GgG > 0$, and also the $GgGgG, \cdots$ (math induction). so higher order terms are always positive. That's why $\lambda = \lambda_{perturbed} + O(higherorder)$ and $\lambda_{perturbed} < \lambda$.

2 Experimental-related design and data analysis

2.1 Experimental methods

Experimental strain and conditions *Enterococcus faecalis* strain OG1RF, a Gram-positive bacterium, was cultured overnight in 50% BHI media in 50 ml cell culture tubes. The minimum inhibitory concentration (MIC) of Linezolid was approximately 1.5 µg/ml, and the MIC of Ampicillin was approximately 0.5 µg/ml. Each antibiotic (Linezolid and Ampicillin) was prepared from powder stock and stored at -20 °C. The migration/transfer cycle time was set to 0.25 hours for the homogeneous case and 0.5 hours for the heterogeneous case. Growth rates were determined using a 1:1 ratio of cell culture to a specific drug solution diluted in 50% BHI media. All dilutions and migrations were completed by an OT-2 pipetting robot dispensing into 96-well plates.

Protocol of growth-migration expriment All cultures were grown at 37 °C in 50% BHI media overnight for 18-20h. All experiments were performed in BioLite 96 Well Multidish. For the spatial heterogeneous migration experiment, the same strain was cultivated under two different conditions: 50% BHI media (high growth rate) and 50% BHI media + 8ug/ml Linezolid (low growth rate).

Cells were diluted 1:5 with 50% BHI media and grew in a new 15ml cell culture tube for 45 minutes before transferring to the 96-well plates and starting the first migration.(Mix the media with or without drug with cells 1:1 ratio). Cell migrations were carried out along the columns of the plate, in 12-well-long landscapes. Migrations were performed every 15/30 minutes using the Opentrons OT-2 robot for ~ 8 times. Plates were not shaken during growth. Optical densities were measured after every migration cycle in the plate reader. with 600-nm light. To explore more possibilities, we changed the transfer volumes to the neighboring columns during the migration in order to control the migration rate. We transferred 5, 12.5, 20, 30, 40, 50, 60, 80, 100 ul(with the single well transfer rate) to the neighboring columns in different plates. The spatially averaged growth rate is controlled by the number of drug wells. As for boundaries, after discarding a transfer volume, an equivalent volume of media (either containing the drug or drug-free, depending on the drug condition of the boundary wells) was added to maintain a consistent well volume.

2.2 Discrete dynamical equation describing the experiment and discretized simplified Fisher-KPP equation

We need to compare the discrete dynamical model that describes the experimental systems with the corresponding discretized form of the 1D Fisher-KPP equation and apply necessary corrections to ensure consistency[1].

According to our experiment protocol, the equation is

$$u_{x,t+\Delta t} = g_{\Delta t}\left(u_{x,t} + b\left(u_{x+\Delta x,t} + u_{x-\Delta x,t} - 2u_{x,t}\right)\right), \tag{S21}$$

where x is the spatial coordinate/well position, t is the cycle number, b is the transfer fraction to a single nearest neighbor, and $g_{\Delta t}(u, x, D)$ describes total growth rate corresponding to the well position x and drug concentration D, also the population density u, i.e. $g_{\Delta t}(u, x, D)$ is the product of the per capita growth rate by the dose-response curve and the population density. Upon linearization $g_{\Delta t}(u)$ can be written as:

$$g_{\Delta t}(u) = e^{g\Delta t},$$

which corresponds to exponential growth at rate g in the incubation time Δt per cycle.

This is an approximation because, in the experiments, we transfer sequentially from well 1 to well 12 and then in reverse order. However, we approximate this as a simultaneous transfer, thereby ignoring the difference between simultaneous and sequential transfers. Additionally, the dilution effect at the boundary wells may need to be considered. After the transfer, to create the deleterious boundary condition, we still need to take out bV ml liquid with bacteria and add bV ml liquid with fresh media(or with drug). Let's assume that the cell density before this operation is u, so the final cell density will be $\frac{1-b}{1+b}u$. Then the effective transfer rate for the boundary wells $b_{edge,eff} = 1 - \frac{1-b}{1+b} \approx 2b$ when b is small.

Using the finite-difference method and discretize the continuous 1D Fisher-KPP equation, we get

$$u_{x,t+\Delta t} = u_{x,t}e^{g_{eff}\Delta t} + \beta_{eff}\frac{\Delta t}{\Delta x^2}\left(u_{x+\Delta x,t} + u_{x-\Delta x,t} - 2u_{x,t}\right) \tag{S22}$$

Comparing eq S21 and eq S22 gives us the effective growth and diffusion rates in the continuous model expressed in terms of experimental parameters as follows:

$$g_{eff} = g,$$

$$\beta_{eff} = b_{eff}\frac{\Delta x^2}{\Delta t}\left(1 + g_{eff}\Delta t\right),$$

where ($\Delta t = 1$ **cycle** $= 0.25$h or 0.5 h, $\Delta x = 1$ well, and $g = \langle g \rangle$ for the homogeneous case and $g = g(x, D)$ for the heterogeneous case. This results in a 'growth-dependent' effective diffusion rate, which unfortunately leads to an iterative, implicit formulation. For simplicity, we also let $g = g(x, D) \approx \langle g \rangle$ for the heterogeneous case. b_{eff} is set to be b or $2b$ as an approximation, due to the effect of boundary well dilution.

To get the largest eigenvalue, the operator of eigenvalue function can also be discretized as a growth-diffusion matrix and numerically evaluated. Rewrite the eigenvalue function in a discrete version with M positions

$$\beta_{disc}u_{i-1} - 2\beta_{disc}u_i + \beta_{disc}u_{i+1} + g_iu_i = \lambda_iu_i$$

where i is the ith spatial position, λ_i is the ith eigenvalue and u_i the ith eigenstate, $k = 1, 2, ...M$; $\beta_{disc} = \beta(M-1)^2/L^2$ In a matrix form we have

$$(\beta_{disc}\mathcal{L} + G)u_\lambda = \lambda u_\lambda$$

where $\mathcal{L} = \begin{bmatrix} -2 & 1 & & & \\ 1 & -2 & 1 & & \\ & \ddots & \ddots & \ddots & \\ & & 1 & -2 & 1 \\ & & & 1 & -2 \end{bmatrix}$ and $G = \begin{bmatrix} \ddots & & \\ & g_i & \\ & & \ddots \end{bmatrix}$. $\mathcal{L}$ is a M by M diffusion matrix and G is a M by M diagonal growth matrix.

2.3 Response determination

The collective response of the bacterial population can either be growing or declining. In the model the criterion of population growing is the largest eigenvalue $\lambda_0 > 0$. For experiment data, the direct way is to compare the total cell density at the final time T and the total cell density at the beginning,

$$\Delta = \langle OD \rangle_F - \langle OD \rangle_0 = \frac{1}{L}\left(\sum_i^L u_i(T) - \sum_i^L u_i(0)\right)$$

To avoid the boundary well effect when drugs are added to supply both boundary wells, a new threshold $\delta_D < 0$ is introduced, ensuring that $\Delta > \delta_D$. Similarly, when media is added, another threshold $\delta_M > 0$ is applied so that $\Delta > \delta_M$. This criterion was used for generating experimental phase diagrams in the main text.

2.4 Growth estimation

The model provides three key parameters for tuning: the growth rate $g(D(x))$, the migration rate β, and the system size L (or 2 effective parameters the growth rate $g(D(x))$ and the boundary diffusion effect $\frac{\beta}{L^2}$). In experimental setups, these correspond to the drug concentration in each well $D(x)$, the transfer rate b, and the number of wells L, respectively. The transfer rate and number of wells can be predetermined before the experiments commence. Once the drug-modulated growth rates are estimated, the system effectively operates without any free parameters, enabling a direct comparison between the model and experimental results.

For a general estimation of bacterial growth, cultures are incubated under specified conditions for several hours, with the growth rate g calculated as follows:

$$g = \frac{1}{T} \ln \frac{u_f}{u_0} = \frac{1}{T} \ln \frac{OD_{\text{final}}}{OD_0}$$

where T represents the duration of the growth period, u_f is the final cell density, and u_0 is the initial cell density, typically measured as optical density (OD). For the growth modulation by the bacteriostatic drug Linezolid (LZD), T is set to 2 hours for homogeneous condition, or 4 hours for heterogeneous condition. In the case of the bactericidal drug Ampicillin (AMP), bacteria are first grown in a drug-free environment for hours, after which AMP is introduced, leading to growth until a significant decrease in OD is observed. The growth rate g and death rate d are subsequently estimated using the method described in [4]. Results are shown in Figure C.

2.5 Simulation

The *PyPDE* Python package is utilized for simulations. *PyPDE* is a Python library designed for solving systems of hyperbolic or parabolic partial differential equations. The simulation closely mirrors the experimental setup. Equation 1 in the main text is discretized into Equation S22 as previously discussed, with a typical system size of $L = 12$. The simulations generally consist of 8 cycles of growth and migration, with parameters selected as outlined earlier.

2.6 Growth validation

Although the growth rate experiments are conducted under the same conditions as the growth-migration experiments, discrepancies may still arise. To further

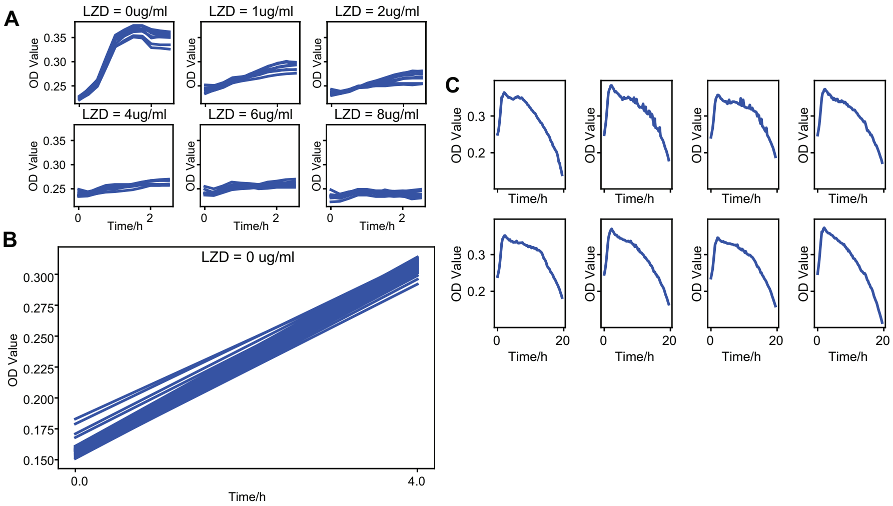

Figure C: **Growth curves of Homo LZD, Hetero LZD, Hetero AMP. A, B.** Growth curves driven by the bacteriostatic drug LZD, with 8 replicates. In panel A, continuous OD curves under 6 different drug concentrations are plotted. In panel B, only the initial OD and final OD were measured. **C.** Panel C shows 8 individual replicates where bacteria were grown in a drug-free environment, followed by the addition of AMP after 2.5 hours.

validate our growth rate estimation, we use the CH/CL experimental data as a representative example(See Figure E, F) to assess the drug-free growth rate. Reasonable values for g are selected within the range of 0 to 0.32 per hour, and the loss is calculated by comparing the data from all 90 experiments with the corresponding simulation results(See Figure D). Despite the overall high loss, our experimentally determined drug-free growth rate $g_{0,exp} = 0.1645$ closely aligns with the optimal rate $g_{0,opt} = 0.17$ identified from the loss landscape. To illustrate, we selected a random example $g_{0,rnd} = 0.0684$ of g to demonstrate the variation in simulation outcomes(See Figure G, H) and compare with the experimental results(See Figure E, F). The optimal growth rate $g_{0,opt} = 0.17$ determined by the loss landscape was also selected to simulate the dynamics(See Figure I, J) and for comparison. Simulation results (Figure I, J) generated with $g_{0,opt} = 0.17$ are closer to the experimental data (see Figure E, F) than the simulations using $g_{0,rnd} = 0.0684$ (see Figure G, H).

3 Simulations and repeated experiments support spatial drug arrangement effect

Detailed evidence has been provided in the main text to demonstrate that different spatial drug arrangements lead to divergent response outcomes. Repeated

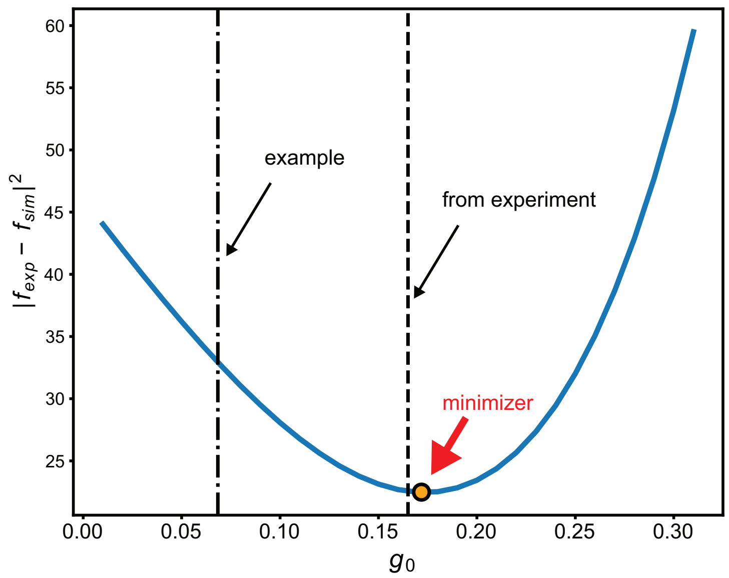

Figure D: **Loss landscape with drug-free growth rate g_0 as the tunable parameter.** The experimentally measured drug-free growth rate is very close to the optimal growth rate shown in this landscape. $g_{0,exp} = 0.0684, g_{0,rnd} = 0.1645, g_{0,opt} = 0.17$.

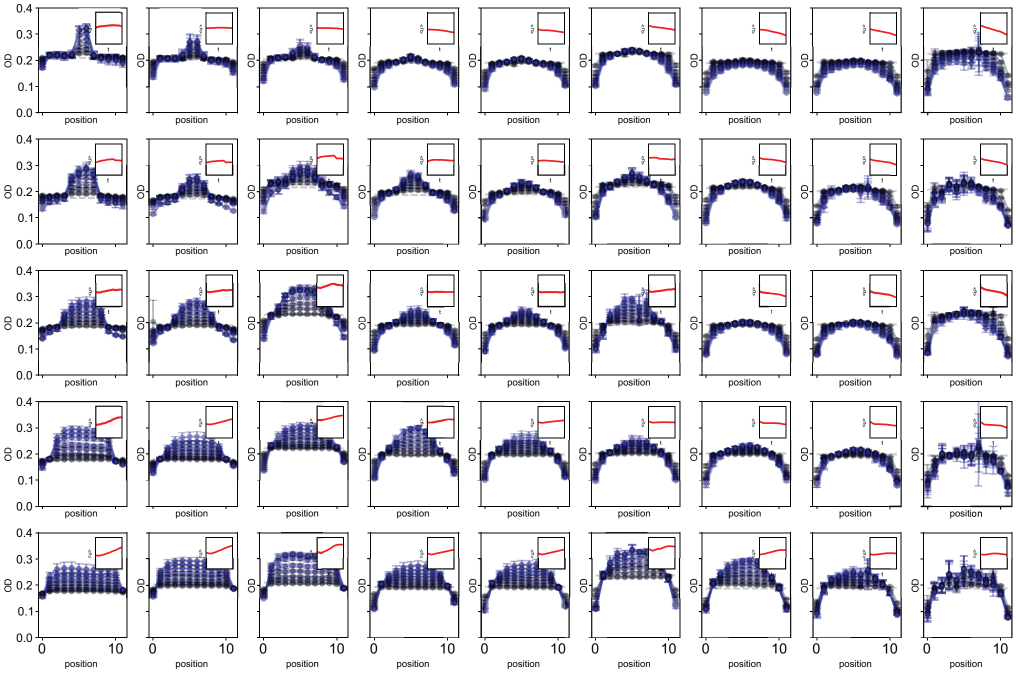

Figure E: **Experimental result of spatial-temporal dynamics across all conditions under the CH arrangement, with experimentally measured drug-free growth rate** $g_{0,exp} = 0.1645$**.** The inset shows the change in spatially averaged population density, $\langle u \rangle$, over time. The dashed line represents the initial optical density (OD). Each curve is the average of eight technical replicates, with error bars corresponding to ± 1 standard deviation of 8 technical replicates.

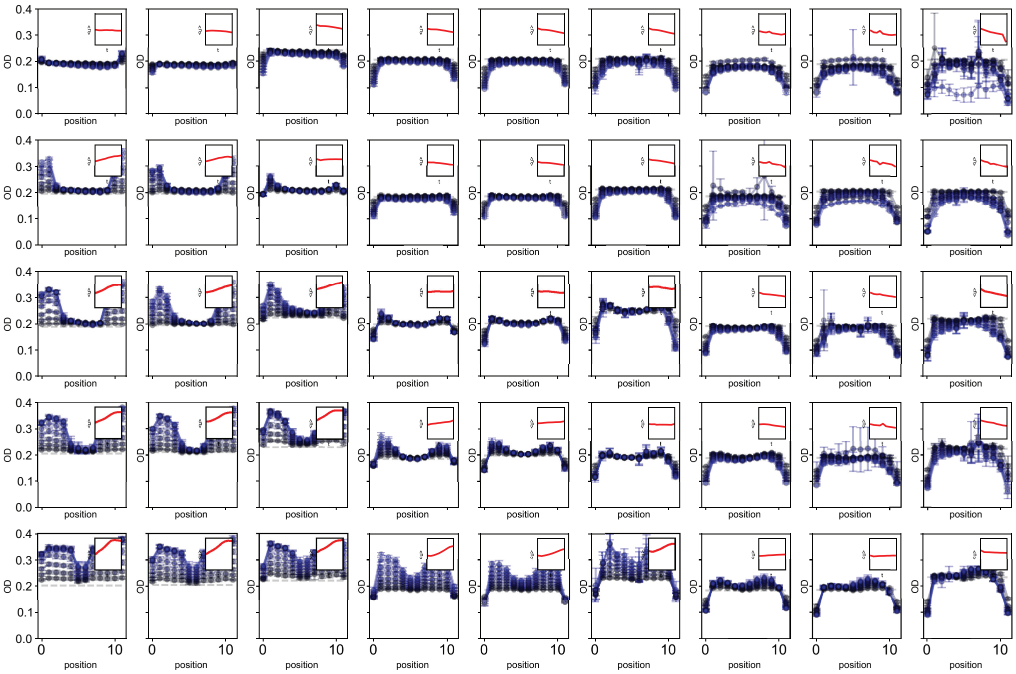

Figure F: **Experimental result of spatial-temporal dynamics across all conditions under the CL arrangement, with experimentally measured drug-free growth rate** $g_{0,exp} = 0.1645$**.** The inset shows the change in spatially averaged population density, $\langle u \rangle$, over time. The dashed line represents the initial optical density (OD). Each curve is the average of eight technical replicates, with error bars corresponding to ± 1 standard deviation of 8 technical replicates.

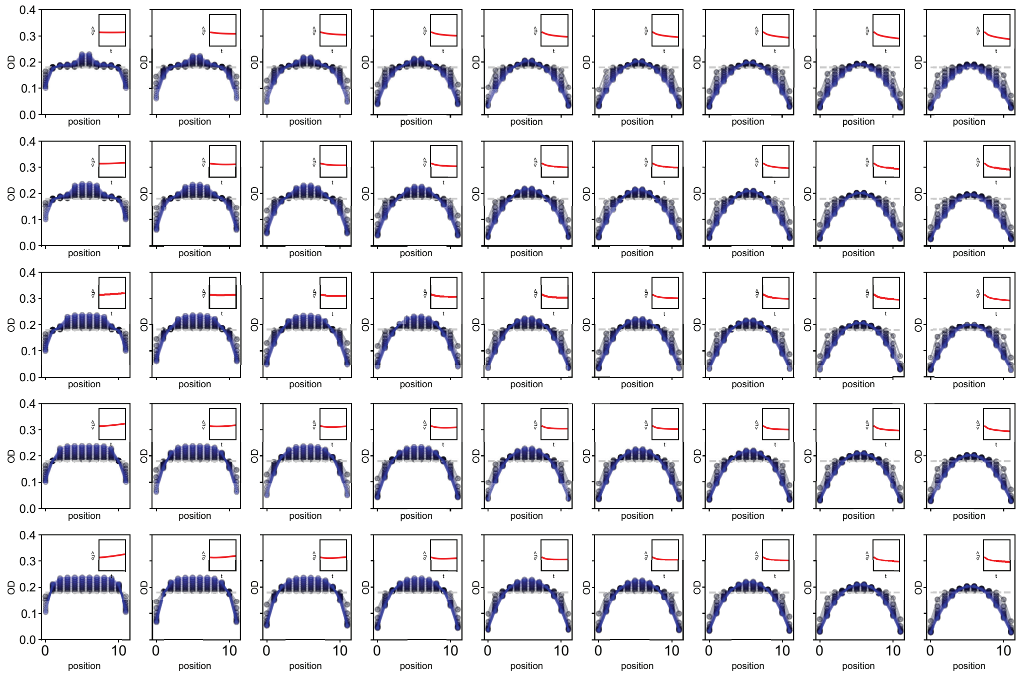

Figure G: **Simulation of spatial-temporal dynamics across all conditions under the CH arrangement, random sampled drug-free growth rate $g_{0,rnd} = 0.0684$.** The inset shows the change in spatially averaged population density, $\langle u \rangle$, over time. The dashed line represents the initial optical density (OD).

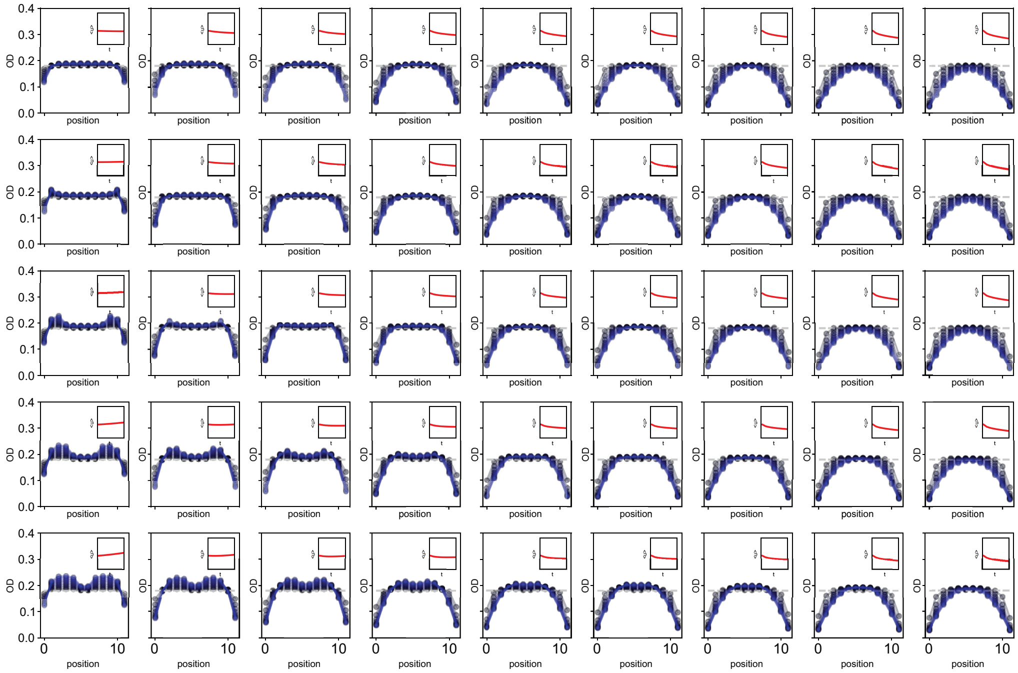

Figure H: **Simulation of spatial-temporal dynamics across all conditions under the CL arrangement, random sampled drug-free growth rate** $g_{0,rnd} = 0.0684$**.** The inset shows the change in spatially averaged population density, $\langle u \rangle$, over time. The dashed line represents the initial optical density (OD).

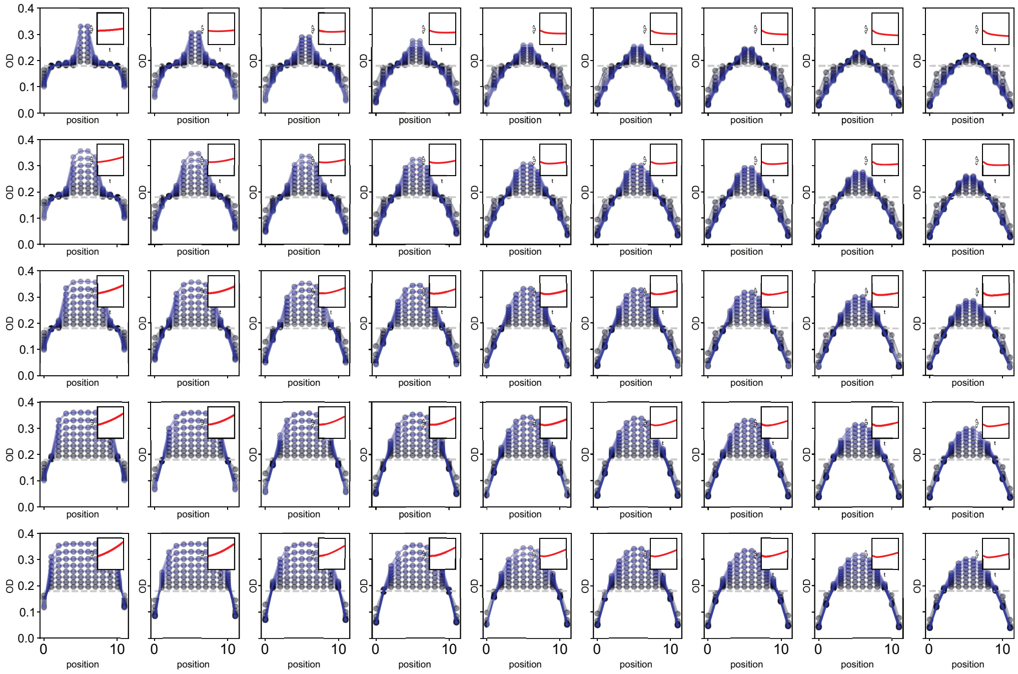

Figure I: **Simulation of spatial-temporal dynamics across all conditions under the CH arrangement, optimal drug-free growth rate $g_{0,opt}$ = 0.17.** The inset shows the change in spatially averaged population density, $\langle u \rangle$, over time. The dashed line represents the initial optical density (OD).

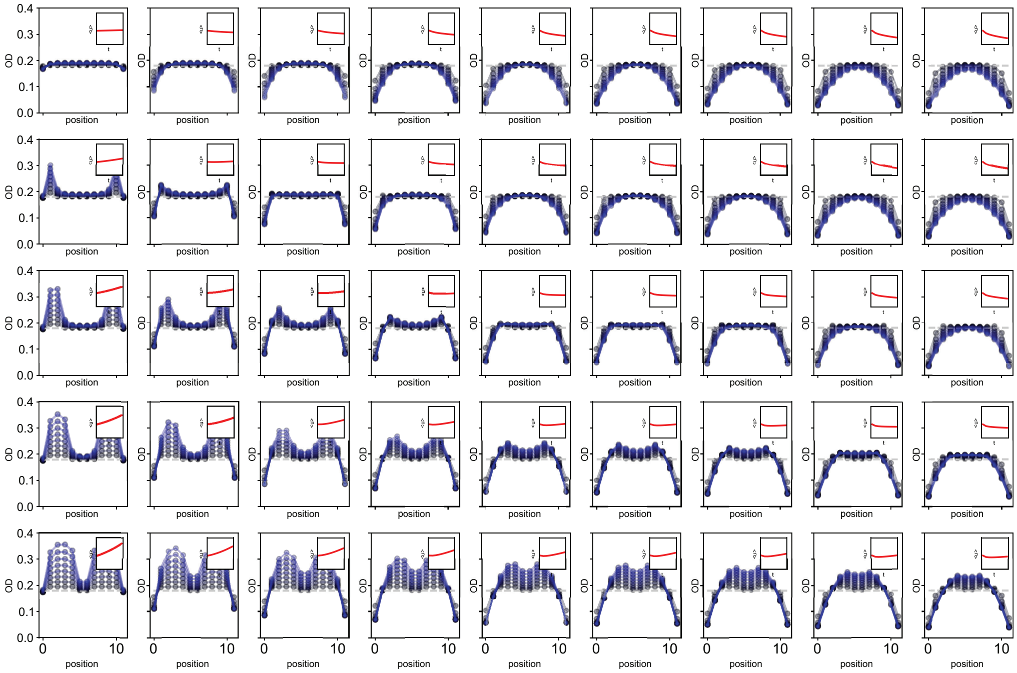

Figure J: **Simulation of spatial-temporal dynamics across all conditions under the CL arrangement, optimal drug-free growth rate** $g_{0,opt} = 0.17$**.** The inset shows the change in spatially averaged population density, $\langle u \rangle$, over time. The dashed line represents the initial optical density (OD).

endpoint experiments and simulations further support this finding. For the initial 6 spatial arrangements, a repeated endpoint experiment was conducted, and despite some discrepancies with Figure 3D in arrangements I and IV, these results, together with the simulation, show strong qualitative agreement (Figure KA, bottom panel). The simulated temporal dynamics(Figure KB) also align well with Figure 3B in the main text. In the experimental phase diagram for CH and CL spatial arrangements, endpoints were remeasured with minimal discrepancies(See Figure L). For different spatial arrangements within a ring structure, the simulation from Figure M shows good agreement with Figure 6B.

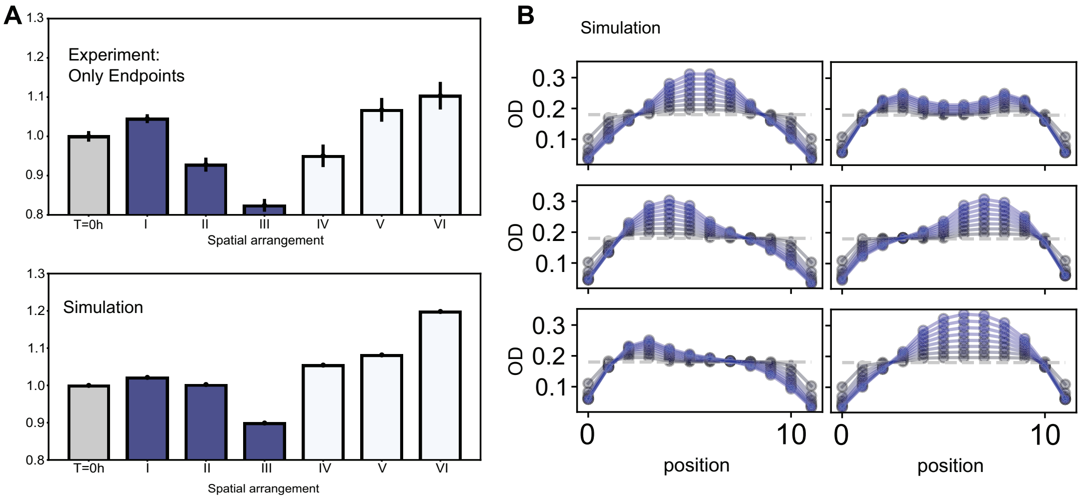

Figure K: **Repeated endpoint experiment of 6 different spatial arrangements, and the simulation results.** Panel A shows a comparison between endpoint experiment barplot (averaged over 8 technical replicates with the error bars corresponding to standard deviation) and the result from simulation. Panel B is simulation of temporal dynamics.

4 Discussion on short-term growth rate, long-term growth rate, and largest eigenvalue λ_0

Although the largest eigenvalue is used in the main text to determine whether the bacterial population is increasing or declining, discrepancies may still arise between the short-term growth rate observed in our short-term diffusion experiments and the largest eigenvalue, which typically represents the long-term

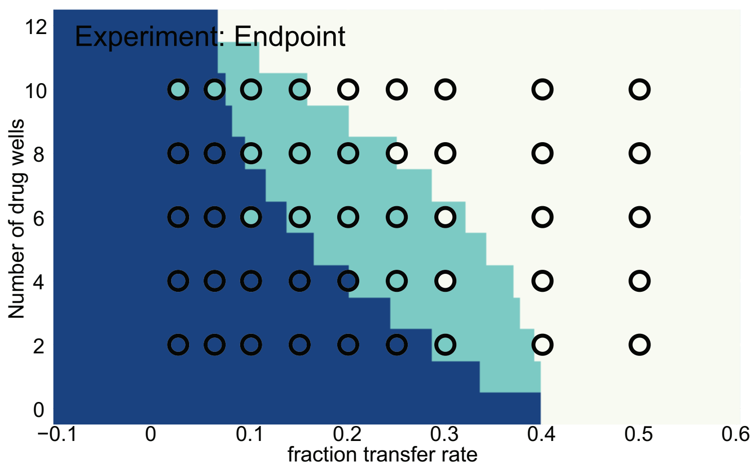

Figure L: **Experimental phase diagram under CH and CL spatial arrangements, only endpoint (independent replicate).**

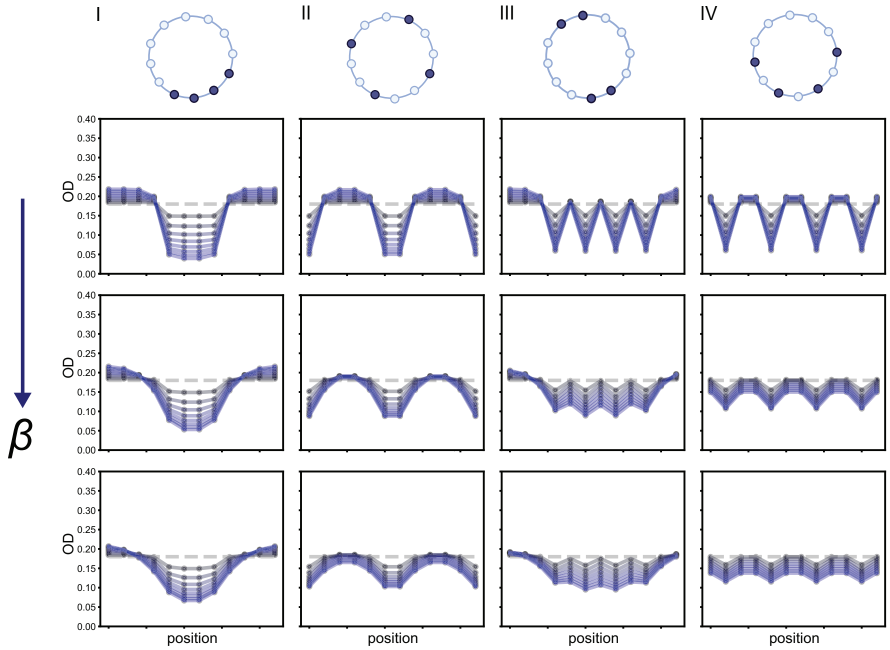

Figure M: **Simulation of temporal dynamics with a ring structure, for 4 spatial arrangements in 3 different migration regimes.** $\beta \approx b\frac{\Delta x^2}{\Delta t} = 0.05, 0.4, 0.8$**well/hour.**

growth rate as time approaches infinity. To explore the relationship between the short-term growth rate, long-term growth rate, and largest eigenvalue, we conducted simulations using all CL spatial arrangements to obtain growth rates at different cycle times. The results are presented below(See Figure N).

Additionally, we compared the perturbation approximation with the numerically solved largest eigenvalue(See Figure O).

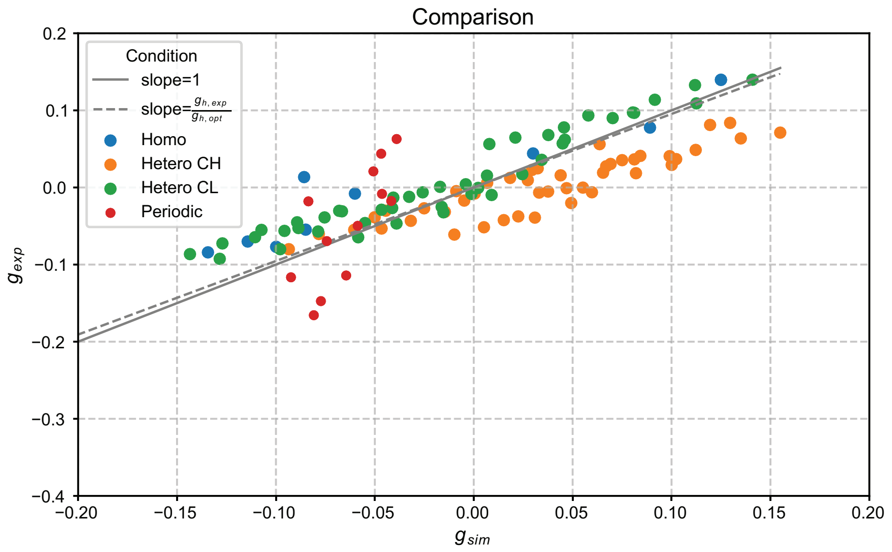

Figure N: **Comparison between simulated short-term growth rate and experimental short-term growth rate.** Growth rates under different conditions - Homo, Hetero CH, Hetero CL, Periodic - are scattered. The solid line represents the identity line with an intercept of 0 and a slope of 1 while the dashed line is a ratio between growth rates estimated from experiments and from the optimal point of the loss landscape. The experimental short-term growth rates match with simulated short-term growth rates well.

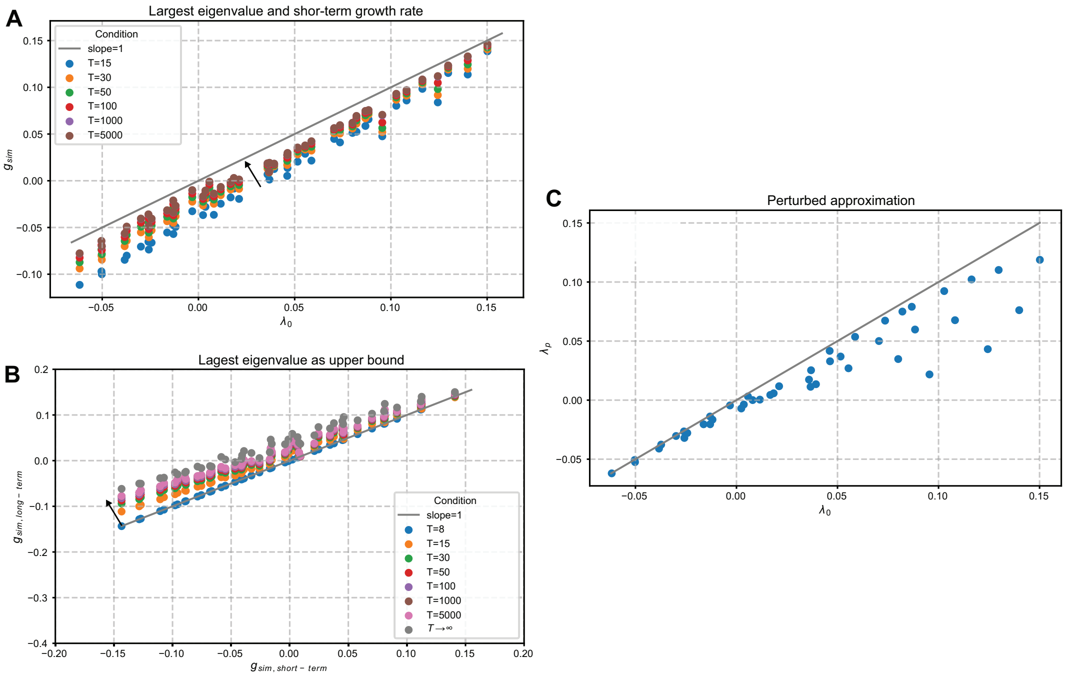

Figure O: **Comparison between short-term growth rate, long-term growth rate, largest eigenvalue, and perturbation approximation.** The solid line represents the identity line with an intercept of 0 and a slope of 1. Panel A shows that as cycle times increase, the short-term growth rate is asymptotically approaching the largest eigenvalue - the largest eigenvalue is effectively the long-term growth rate after infinite time; Panel B shows that short-term growth rate is always lower than long-term experiments; Panel C shows that the perturbation approximation matches with real eigenvalues well when the original eigenvalue is small.

5 Discussion on drug diffusion effect

In our experimental set-up, we considered only growth and migration and removed the dilution step used in other spatial growth–migration experiments performed with pipetting robots, such as range–expansion studies[1]. This choice increases efficiency for parameter sweeps and avoids long-term experiments. Including a dilution step after each migration cycle would substantially increase the time required for bacteria to grow back to a measurable density. However, omitting dilution raises the possibility that drug may also “diffuse” through pipetting during liquid transfer. Here, we use simulations to evaluate the impact of drug diffusion and show that it does not affect our qualitative con-

clusions. Thus, although this diffusion exists, we can effectively ignore it, and time-dependent changes in drug concentrations do not affect bacterial densities on the timescales used in this study. We examine drug diffusion under three conditions: (a) spatial drug heterogeneity with absorbing boundaries; (b) spatial drug heterogeneity with periodic boundaries; and (c) spatial drug homogeneity with absorbing boundaries. For both LZD and AMP, we only measured high growth rates at $D = 0$ μg/ml and low growth rates at $D = 8$ μg/ml (LZD) or $D = 100$ μg/ml (AMP), so interpolation was required to estimate $g(D)$ between these values. We used linear interpolation in all simulations.

Heterogeneity with absorbing boundaries We first fix the bacterial diffusion rate β and vary the drug diffusion rate β_D to examine how final bacterial densities depend on drug diffusion. Using the CL arrangement with 6 drug wells as an example (Figure P), we observe that although the final drug distributions differ substantially across drug diffusion rates (as shown by the time-varying drug profiles), the final bacterial density profiles are nearly identical. This is also reflected in the temporal evolution of bacterial spatial heterogeneity. These results demonstrate that, within 8 migration cycles, drug diffusion does not qualitatively alter population dynamics. Similar results were obtained for the CH arrangement with 6 drug wells (Figure Q).

Heterogeneity with periodic boundaries Next, we consider periodic boundary conditions and examine two patterns (I and III). For Pattern I, fixing the bacterial diffusion rate β and varying the drug diffusion rate produces only small differences in final bacterial densities (Figure R). However, for Pattern III, the differences are larger (Figure S). This suggests the possibility of a time-delay or "frozen" growth effect for AMP: although the drug diffuses and alters its spatial distribution over time, the effective growth rates may remain approximately fixed over the 8 time steps considered. This behavior is not captured by our current minimal model. Nonetheless, even under these conditions, the qualitative behavior (growth versus decline) remains consistent with our main conclusions.

Homogeneity with absorbing boundaries Finally, we investigate drug diffusion under spatially homogeneous drug concentrations, comparing no drug ($D = 0$ μg/ml), low drug ($D = 1$ μg/ml), and high drug ($D = 8$ μg/ml). As expected, for $D = 0$ μg/ml, all results are nearly identical across β_D, with only minor numerical fluctuations (Figure T). For $D = 1$ μg/ml, the uniform drug distribution gradually develops a "bell-shaped" profile as β_D increases, but the final bacterial densities remain very similar (Figure U). For $D = 8$ μg/ml, the final bacterial densities show somewhat larger differences, yet still qualitatively match each other within 8 cycles (Figure V).

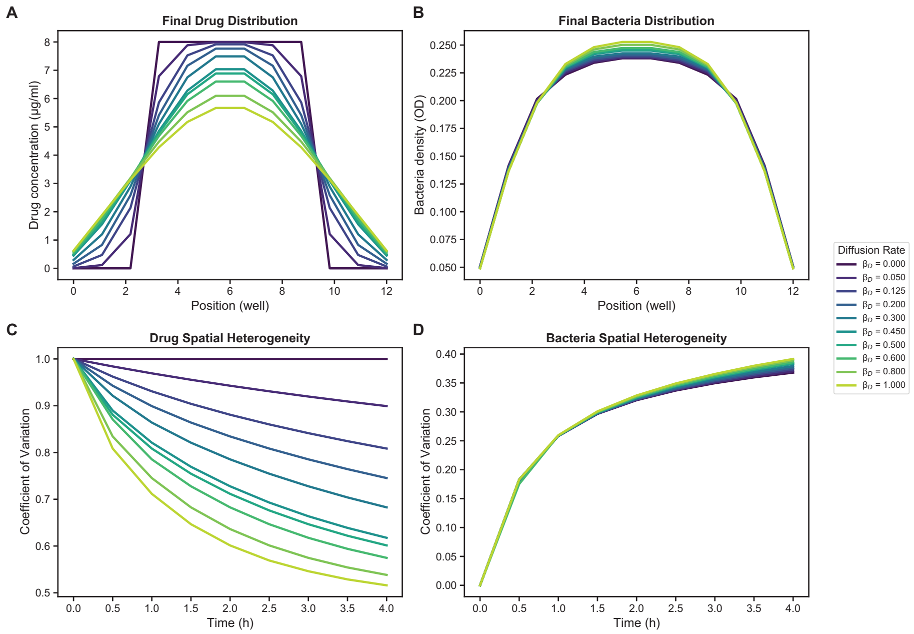

Figure P: **Endpoint simulation results of drug and bacteria diffusion dynamics in spatially heterogeneous environments with central high-drug region (or CL in terms of growth rate).** **A.** Final spatial distribution of drug concentration across different drug diffusion rates (β_D). **B.** Final spatial distribution of bacterial density (OD). **C.** Drug spatial heterogeneity over time measured by coefficient of variation. **D.** Bacteria spatial heterogeneity over time across different drug diffusion rates. Different colors of lines represent different drug diffusion coefficients (β_D) ranging from 0 to 1.0 well$^2 \cdot$h^{-1}. Initial conditions: uniform bacterial density (OD = 0.18), central high-drug band (D = 8 ug/ml in wells 4-9, D = 0 elsewhere). Simulation parameters: system size L = 12 wells, duration time T = 4 h, bacteria diffusion coefficient $\beta_u = 0.8$ h^{-1}.

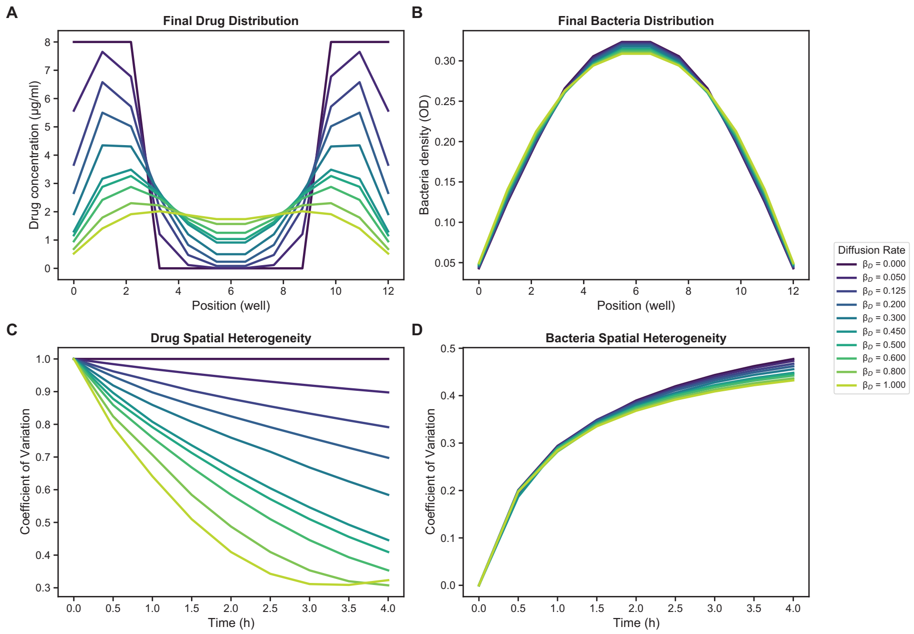

Figure Q: **Endpoint simulation results of drug and bacteria diffusion dynamics in spatially heterogeneous environments with central low-drug region (or CH in terms of growth rate).** **A.** Final spatial distribution of drug concentration across different drug diffusion rates (β_D). **B.** Final spatial distribution of bacterial density (OD). **C.** Drug spatial heterogeneity over time measured by coefficient of variation. **D.** Bacteria spatial heterogeneity over time across different drug diffusion rates. Different colors of lines represent different drug diffusion coefficients (β_D) ranging from 0 to 1.0 well$^2 \cdot$h^{-1}. Initial conditions: uniform bacterial density (OD = 0.18), central high-drug band (D = 0 ug/ml in wells 4-9, D = 8 ug/ml elsewhere). Simulation parameters: system size L = 12 wells, duration time T = 4 h, bacteria diffusion coefficient $\beta_u = 0.8$ h^{-1}.

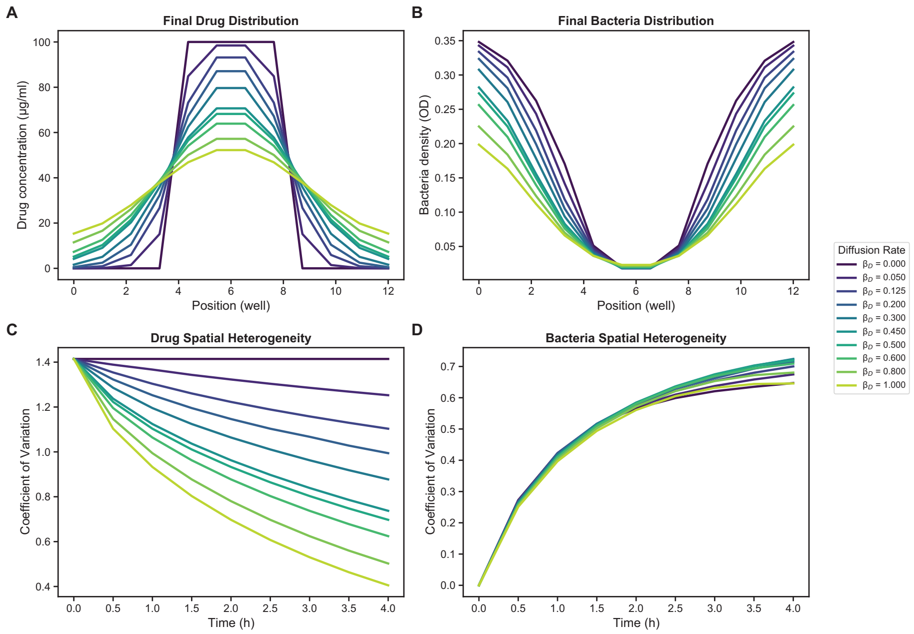

Figure R: **Endpoint simulation results of drug and bacteria diffusion dynamics under periodic boundary conditions with contiguous high-drug region (Pattern I). A.** Final spatial distribution of drug concentration across different drug diffusion rates (β_D). **B.** Final spatial distribution of bacterial density (OD). **C.** Drug spatial heterogeneity over time measured by coefficient of variation across different drug diffusion rates. **D.** Bacteria spatial heterogeneity over time. Different colors of lines represent different drug diffusion coefficients (β_D) ranging from 0 to 1.0 well$^2 \cdot$ h^{-1}. Initial conditions: uniform bacterial density (OD = 0.18), contiguous high-drug region (D = 100 ug/ml in wells 5-8, D = 0 elsewhere), periodic boundary condition. Simulation parameters: system size L = 12 wells, duration time T = 4 h, bacteria diffusion coefficient $\beta_u = 0.8$ h^{-1}.

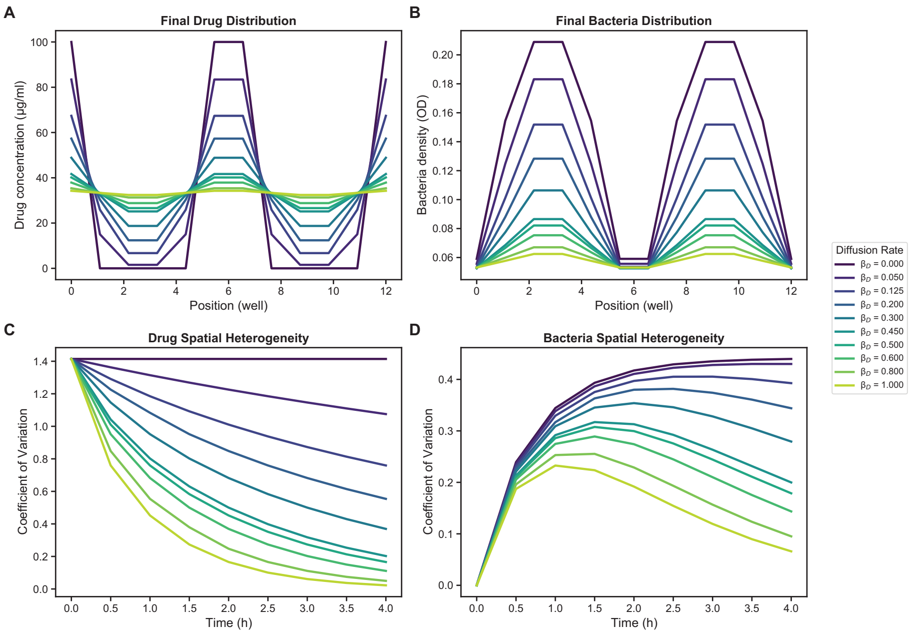

Figure S: **Endpoint simulation results of drug and bacteria diffusion dynamics under periodic boundary conditions with fragmented high-drug pattern (Pattern III). A.** Final spatial distribution of drug concentration across different drug diffusion rates (β_D). **B.** Final spatial distribution of bacterial density (OD). **C.** Drug spatial heterogeneity over time measured by coefficient of variation across different drug diffusion rates. **D.** Bacteria spatial heterogeneity over time. Different colors of lines represent different drug diffusion coefficients (β_D) ranging from 0 to 1.0 well$^2 \cdot$ h^{-1}. Initial conditions: uniform bacterial density (OD = 0.18), fragmented high-drug pattern (D = 100 ug/ml in wells 1, 6, 7, 12, D = 0 elsewhere), periodic boundary conditions. Simulation parameters: system size L = 12 wells, duration time T = 4 h, bacteria diffusion coefficient $\beta_u = 0.8$ h^{-1}.

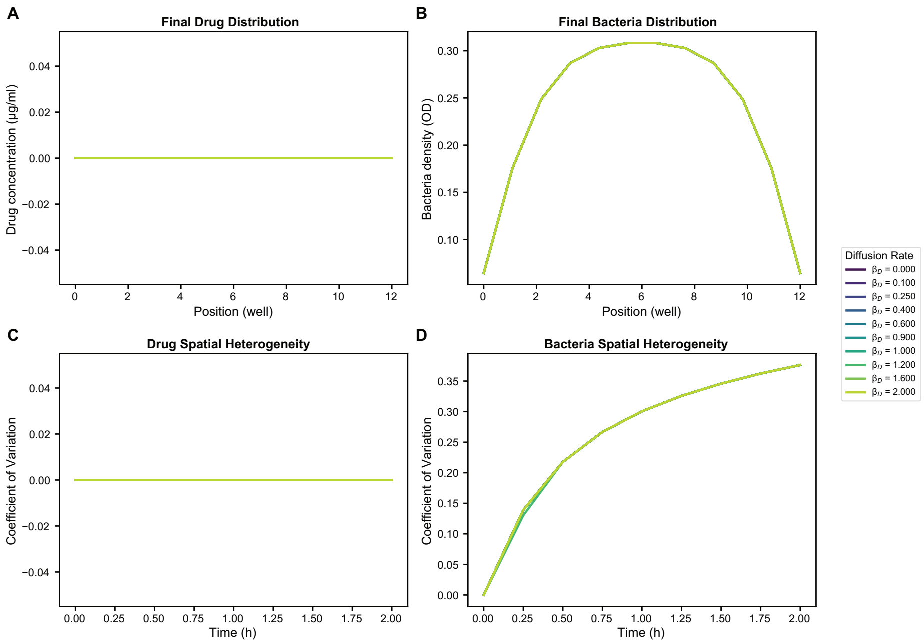

Figure T: **Endpoint simulation results of drug and bacteria diffusion dynamics in spatially homogeneous environments without drug treatment. A.** Final spatial distribution of drug concentration across different drug diffusion rates (β_D). **B.** Final spatial distribution of bacterial density (OD) showing uniform growth in drug-free conditions. **C.** Drug spatial heterogeneity over time measured by coefficient of variation, remaining at zero across all diffusion rates. **D.** Bacteria spatial heterogeneity over time showing minimal variation due to homogeneous initial conditions. Different colors of lines represent different drug diffusion coefficients (β_D) ranging from 0 to 2.0 well$^2 \cdot$ h^{-1}. Initial conditions: uniform bacterial density (OD = 0.18), no drug (D = 0 ug/ml). Simulation parameters: system size L = 12 wells, duration time T = 2 h, bacteria diffusion coefficient $\beta = 1.0$ h^{-1}.

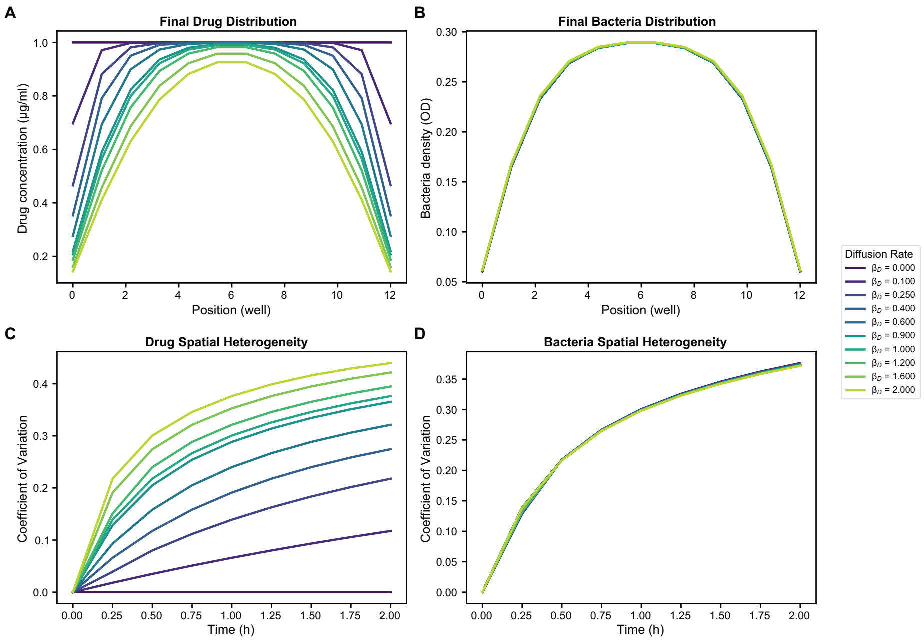

Figure U: **Endpoint simulation results of drug and bacteria diffusion dynamics in spatially homogeneous environments under low drug concentration. A.** Final spatial distribution of drug concentration across different drug diffusion rates (β_D), showing homogenization at high diffusion rates. **B.** Final spatial distribution of bacterial density (OD) demonstrating growth inhibition under uniform low drug exposure (1 ug/ml). **C.** Drug spatial heterogeneity over time measured by coefficient of variation. **D.** Bacteria spatial heterogeneity over time showing minimal variation due to homogeneous initial conditions. Different colors of lines represent different drug diffusion coefficients (β_D) ranging from 0 to 2.0 well$^2 \cdot$h^{-1}. Initial conditions: uniform bacterial density (OD = 0.18), uniform low drug concentration (D = 1 ug/ml). Simulation parameters: system size L = 12 wells, duration time T = 2 h, bacteria diffusion coefficient $\beta_u = 1.0$ h^{-1}.

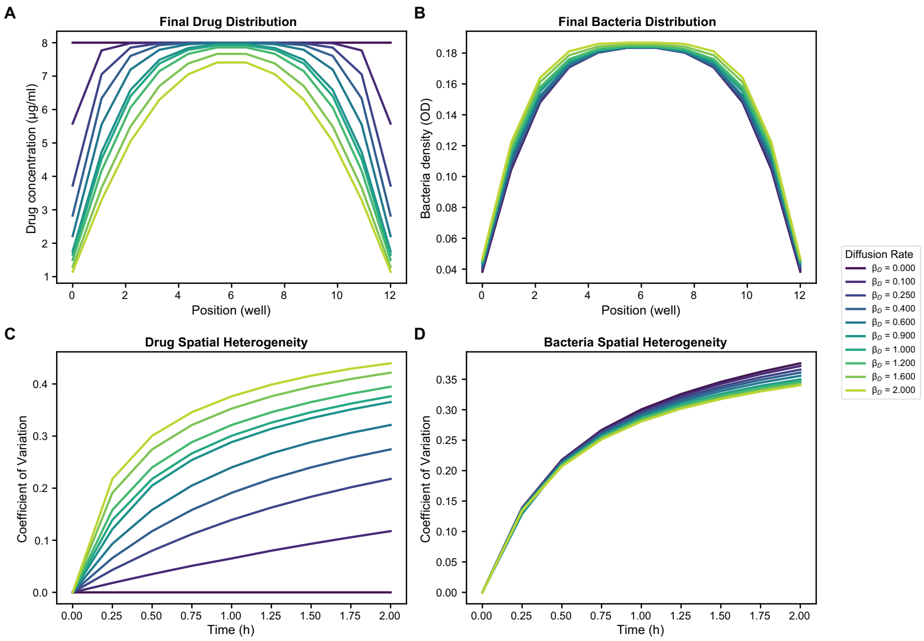

Figure V: **Endpoint simulation results of drug and bacteria diffusion dynamics in spatially homogeneous environments under high drug concentration. A.** Final spatial distribution of drug concentration across different drug diffusion rates (β_D). **B.** Final spatial distribution of bacterial density (OD) showing strong growth inhibition with minimal spatial variation. **C.** Drug spatial heterogeneity over time measured by coefficient of variation. **D.** Bacteria spatial heterogeneity over time showing minimal variation, indicating uniformly suppressed growth across the domain. Different colors of lines represent different drug diffusion coefficients (β_D) ranging from 0 to 2.0 well$^2 \cdot$ h^{-1}. Initial conditions: uniform bacterial density (OD = 0.18), uniform high drug concentration (D = 8 ug/ml). Simulation parameters: system size L = 12 wells, duration time T = 2 h, bacteria diffusion coefficient $\beta_u = 1.0$ h^{-1}.

6 Discussion on active migration with a metabolic cost

In this study, bacterial migration is passive and does not impose a metabolic burden. It is therefore useful to consider an extended condition in which bacteria migrate actively, incurring a metabolic cost. Overall, if all other settings remain the same, including active migration does not alter our core findings regarding the existence of the mixed phase. Below we provide an initial analysis.

Assume (a) active migration introduces an advection term with constant

speed v into the model, and (b) the metabolic cost reduces the drug-induced growth rate by c. The resulting one-dimensional KiSS model becomes

$$\frac{\partial u}{\partial t} = \beta \frac{\partial^2 u}{\partial x^2} - v \frac{\partial u}{\partial x} + (g(D(x)) - c)u, \tag{S1}$$

with absorbing boundaries $u(0,t) = u(L,t) = 0$. A positive v corresponds to active migration toward the right $(+x)$, while negative v corresponds to migration toward the left $(-x)$. We assume the solution has the form $u(x,t) = u_t(t)u_x(x) = u_0 e^{\lambda t}\phi(x)e^{\frac{v}{2\beta}x}$. The first equality follows from separation of variables, and the second introduces an ansatz via a gauge transformation. Substituting this into Eq. S1 and rearranging yields

$$\left(g(D(x)) + \beta \frac{\partial^2}{\partial x^2} - \left(\frac{v^2}{4\beta} + c\right)\right)\phi = \lambda\phi. \tag{S2}$$

For a homogeneous environment, the eigenvalues can be derived exactly:

$$\lambda_k = \langle g \rangle - \left(\frac{v^2}{4\beta} + c\right) - \frac{\beta\pi^2(k+1)^2}{L^2}. \tag{S3}$$

The corresponding eigenvectors are $\phi_k = \sqrt{\frac{2}{L}}\sin\left(\frac{(k+1)\pi x}{L}\right)$, and therefore the full spatial density becomes $u_k = e^{\frac{v}{2\beta}x}\phi_k = \sqrt{\frac{2}{L}}e^{\frac{v}{2\beta}x}\sin\left(\frac{(k+1)\pi x}{L}\right)$. Relative to passive migration, the largest eigenvalue λ_0 is decreased by $\frac{v^2}{4\beta} + c$, and the spatial profile is tilted by $e^{\frac{v}{2\beta}x}$ toward the direction of active movement.

For heterogeneous environments, applying first-order perturbation theory yields:

$$\lambda_0 \approx \lambda_{\text{perturbed}} = \langle g \rangle - \left(\frac{v^2}{4\beta} + c\right) - \frac{\pi^2\beta}{L^2} + \langle \phi_0 | \delta g | \phi_0 \rangle. \tag{S14}$$

Thus, the approximated largest eigenvalue is also decreased by $\frac{v^2}{4\beta} + c$.

From this initial analysis, active migration with metabolic cost introduces two new effects: (a) an additional boundary-diffusion-like term $\frac{v^2}{4\beta}$ arising from advection, and (b) a growth penalty c from metabolic cost. The amplitude of the new boundary diffusion term depends on both active migration speed v and passive migration rate β. Interestingly, increasing β reduces the magnitude of the $\frac{v^2}{4\beta}$ term while simultaneously increasing the usual passive boundary diffusion effect. The metabolic cost c decreases the largest eigenvalue λ_0 directly. In some cases c may depend on v, which would further complicate the analysis.

For homogeneous environments, the classic growth condition depends on both new effects. For heterogeneous environments, the spatial drug heterogeneity term $\langle \phi_0 | \delta g | \phi_0 \rangle$ is unchanged (the tilt term $e^{\frac{v}{2\beta}x}$ does not appear in the perturbation expression). This suggests that the "arrangement-dependent" mixed phase still exists; at least in the limit of large passive migration, the perturbation approximation remains valid. The exact shape of the new mixed phase depends on the combined term $\left(\frac{v^2}{4\beta} + c\right)$.

7 Discussion on gradual concentration change over space

In this study, spatial antibiotic concentrations change in a step-like manner, with only drug wells and drug-free wells, as different community sites are explored. This design was chosen for experimental simplicity: permuting drug versus non-drug wells to create different spatial arrangements is far easier than engineering arbitrary spatial heterogeneities, and it also reduces potential discrepancies introduced by drug diffusion. We emphasize that whether antibiotic concentrations change gradually or discontinuously does not affect the existence of spatial drug arrangement effects or the "arrangement-dependent" mixed-response phase. Using stepwise concentrations such as CH and CL provides a minimal and experimentally tractable way to demonstrate the mixed phase, because we have shown that these two configurations represent extreme spatial arrangements that maximize growth or decline and therefore produce the largest response differences. For a fixed spatially averaged growth rate and boundary diffusion rate, a gradually changing drug concentration profile will not be optimal; its population response will fall between those produced by CH and CL. In other words, step-like profiles allow us to span the full response range, while smoother profiles yield intermediate outcomes. Moreover, step-like spatial drug distributions may be more biologically relevant than gradual ones in certain clustered microenvironments in the human body, such as facial skin pores[5, 6, 7] and gut villi/crypts[8]. Human facial skin contains on the order of 20,000 pores, and the number of gut crypts is approximately 100,000. Chemicals such as antibiotics can remain localized within individual microregions, while bacteria are able to migrate between them. Thus, step-like spatial heterogeneities may represent a natural and relevant limit of the broader class of spatial drug gradients.

References

[1] S. R. Gandhi, E. A. Yurtsev, K. S. Korolev, and J. Gore, "Range expansions transition from pulled to pushed waves as growth becomes more cooperative in an experimental microbial population," *Proceedings of the National Academy of Sciences*, vol. 113, no. 25, pp. 6922–6927, 2016.

[2] C. M. Miller and J. A. Draghi, "Range expansion can promote the evolution of plastic generalism in coarse-grained landscapes," *Evolution Letters*, p. qrad062, 2023.

[3] T. F. A. Freire, Z. Hu, K. B. Wood, and E. Gjini, "Modeling spatial evolution of multi-drug resistance under drug environmental gradients," *PLOS Computational Biology*, vol. 20, no. 5, p. e1012098, 2024.

[4] A. J. Lee, S. Wang, H. R. Meredith, B. Zhuang, Z. Dai, and L. You, "Robust, linear correlations between growth rates and β-lactam–mediated lysis rates,"

Proceedings of the National Academy of Sciences, vol. 115, no. 16, pp. 4069–4074, 2018.

[5] P. M. Maia Campos, M. O. Melo, and D. G. Mercurio, "Use of advanced imaging techniques for the characterization of oily skin," *Frontiers in Physiology*, vol. 10, p. 254, 2019.

[6] J. Claesen, J. B. Spagnolo, S. F. Ramos, K. L. Kurita, A. L. Byrd, A. A. Aksenov, A. V. Melnik, W. R. Wong, S. Wang, R. D. Hernandez, *et al.*, "A cutibacterium acnes antibiotic modulates human skin microbiota composition in hair follicles," *Science translational medicine*, vol. 12, no. 570, p. eaay5445, 2020.

[7] A. Conwill, A. C. Kuan, R. Damerla, A. J. Poret, J. S. Baker, A. D. Tripp, E. J. Alm, and T. D. Lieberman, "Anatomy promotes neutral coexistence of strains in the human skin microbiome," *Cell Host & Microbe*, vol. 30, no. 2, pp. 171–182, 2022.

[8] C. Casteleyn, A. Rekecki, A. Van der Aa, P. Simoens, and W. Van Den Broeck, "Surface area assessment of the murine intestinal tract as a prerequisite for oral dose translation from mouse to man," *Laboratory animals*, vol. 44, no. 3, pp. 176–183, 2010.
